# Supplementary material for: Systematic Review and Meta-Analysis on Prevalence and Antimicrobial Resistance Patterns of Important Foodborne Pathogens Isolated from Retail Chicken Meat and Associated Environments in India
Source: Foods. 2025 Feb 7;14(4):555. doi: 10.3390/foods14040555 (PMC11854295; doi:10.3390/foods14040555)
Supplement: Supplementary file 1 [file foods-14-00555-s001.zip › foods-3373930-supplementary.pdf]

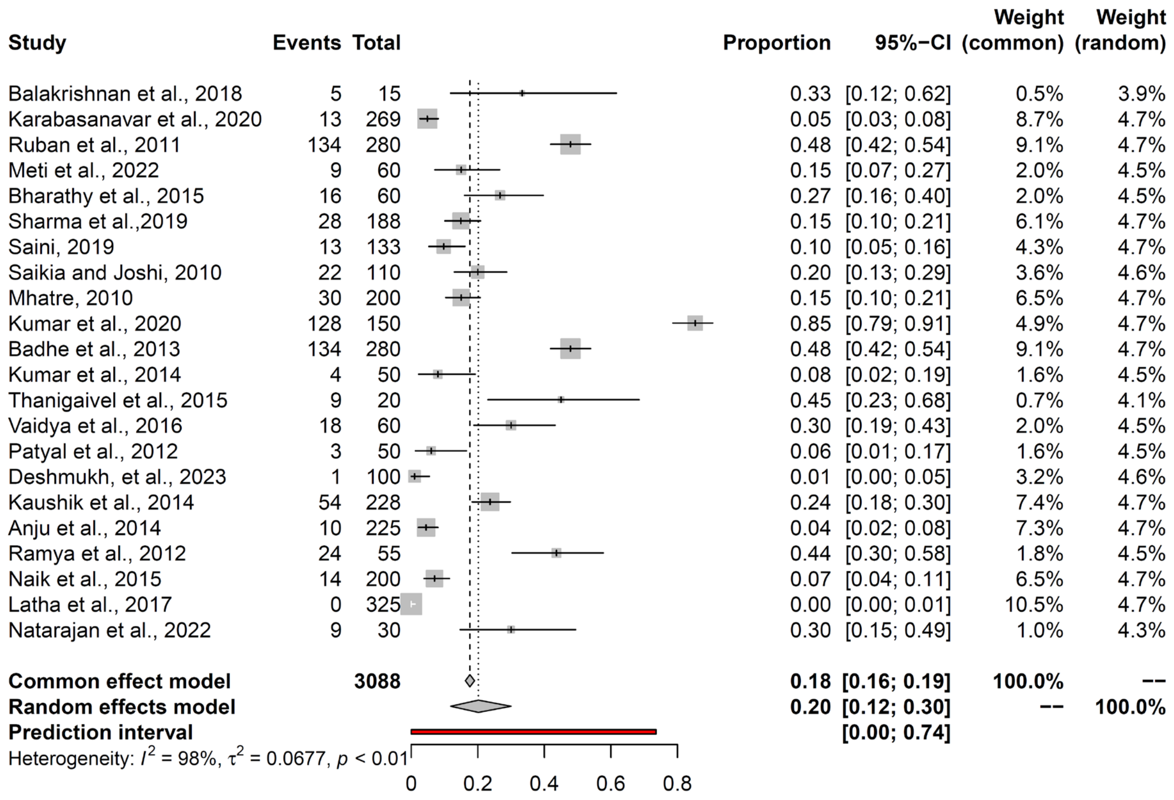

Figure S1; Forest plot depicting the pooled prevalence of *Salmonella* spp. in retail chicken meat

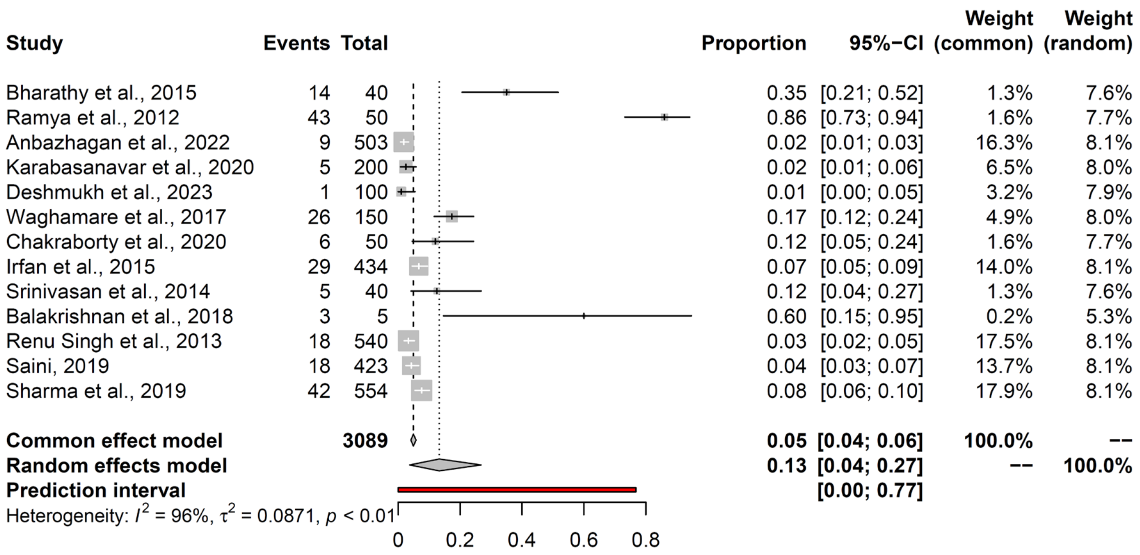

Figure S2; Forest plot depicting the pooled prevalence of *Salmonella* spp. in the chicken-associated environment

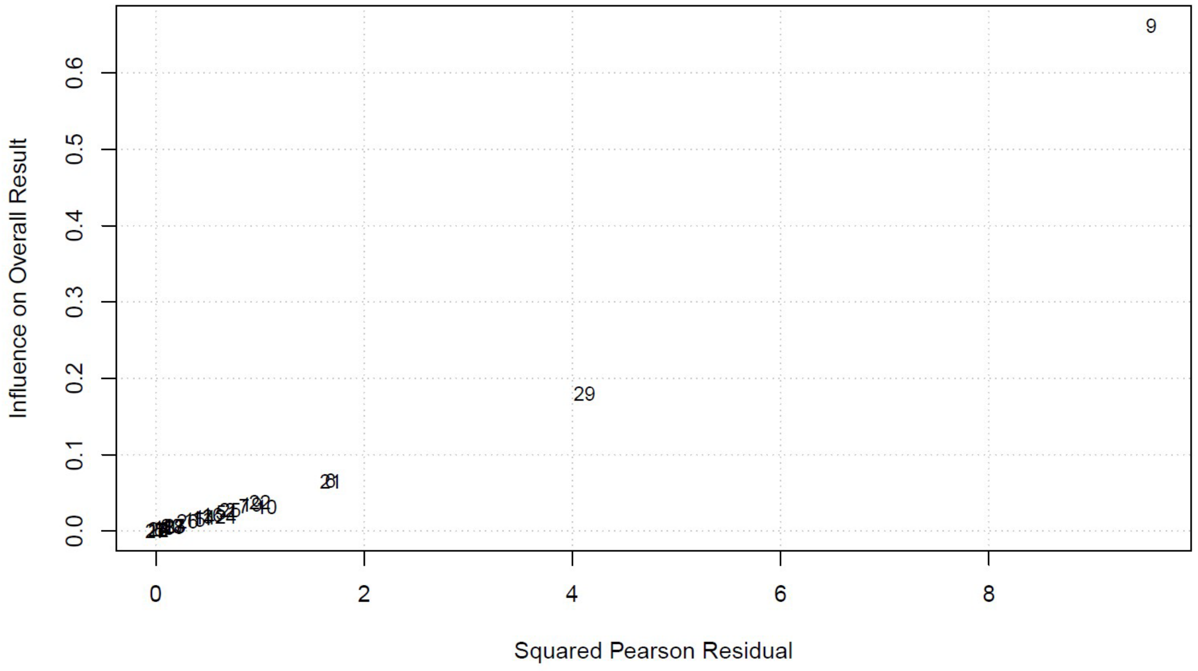

Figure S3; Baujat plot depicting outlier studies for retail chicken meat and associated environment for *Salmonella* spp.

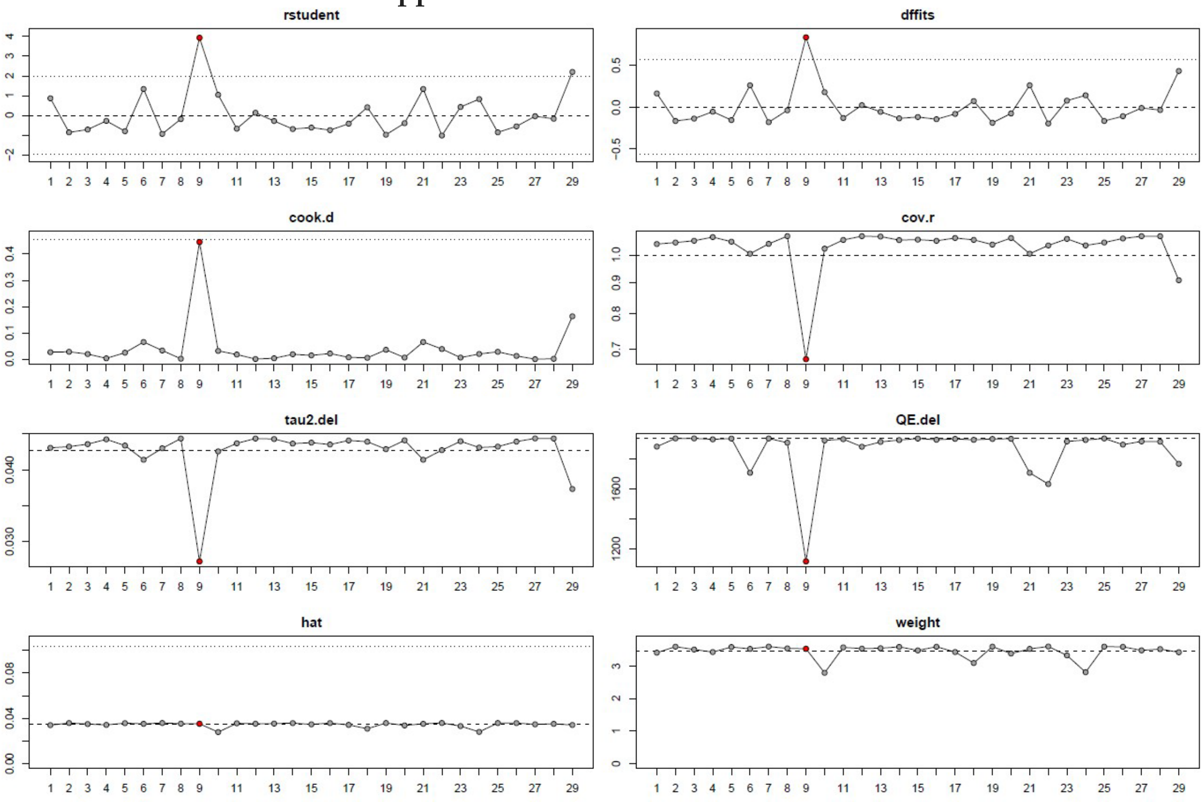

Figure S4; Plot depicting influential studies for retail chicken meat and associated environment for *Salmonella* spp

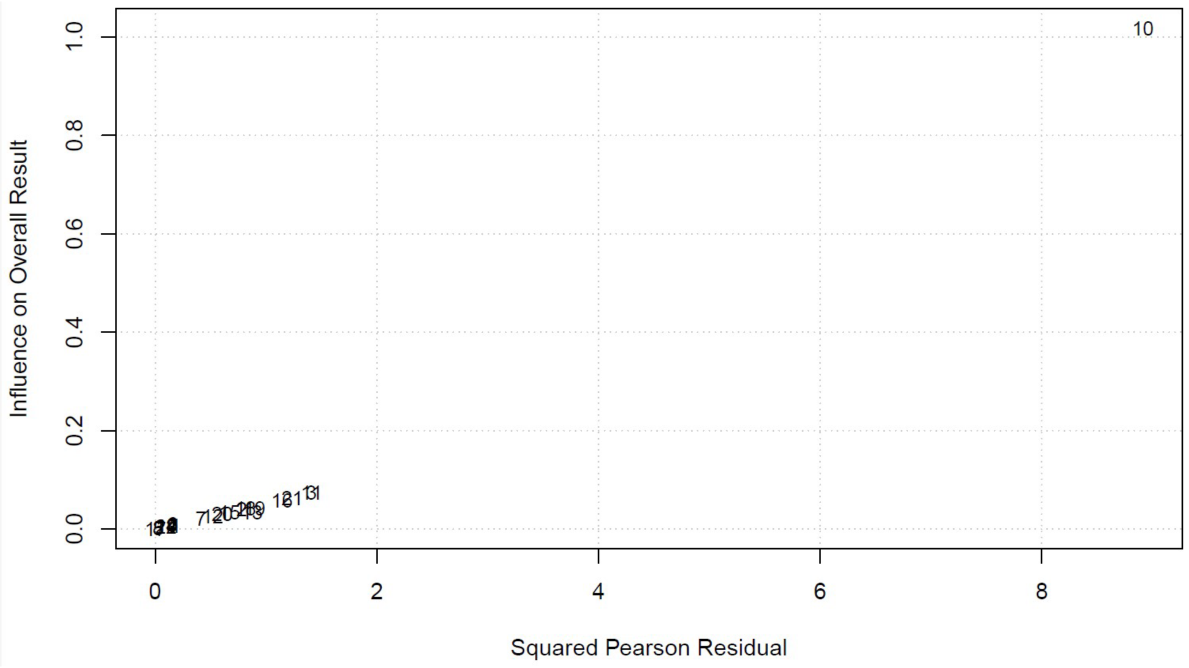

Figure S5; Baujat plot depicting outlier studies for retail chicken meat for *Salmonella* spp.;

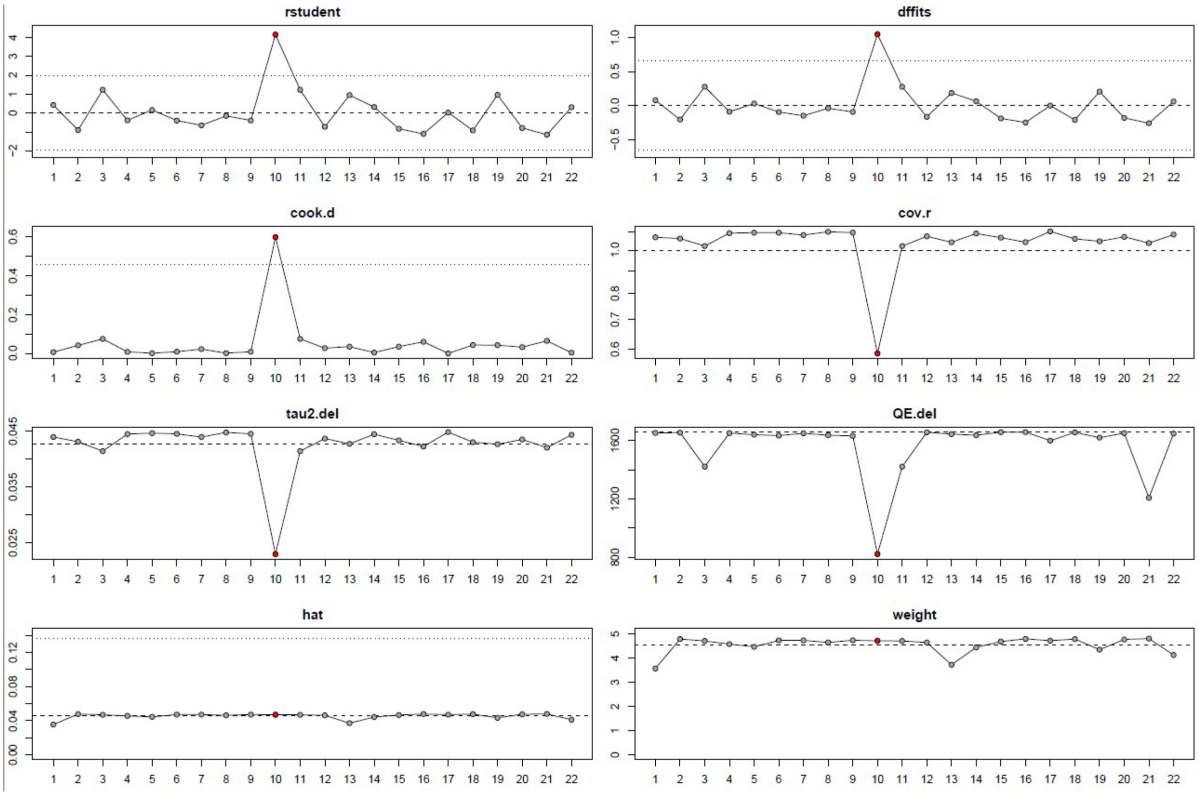

Figure S6; Plot depicting influential studies for retail chicken meat for *Salmonella* spp.

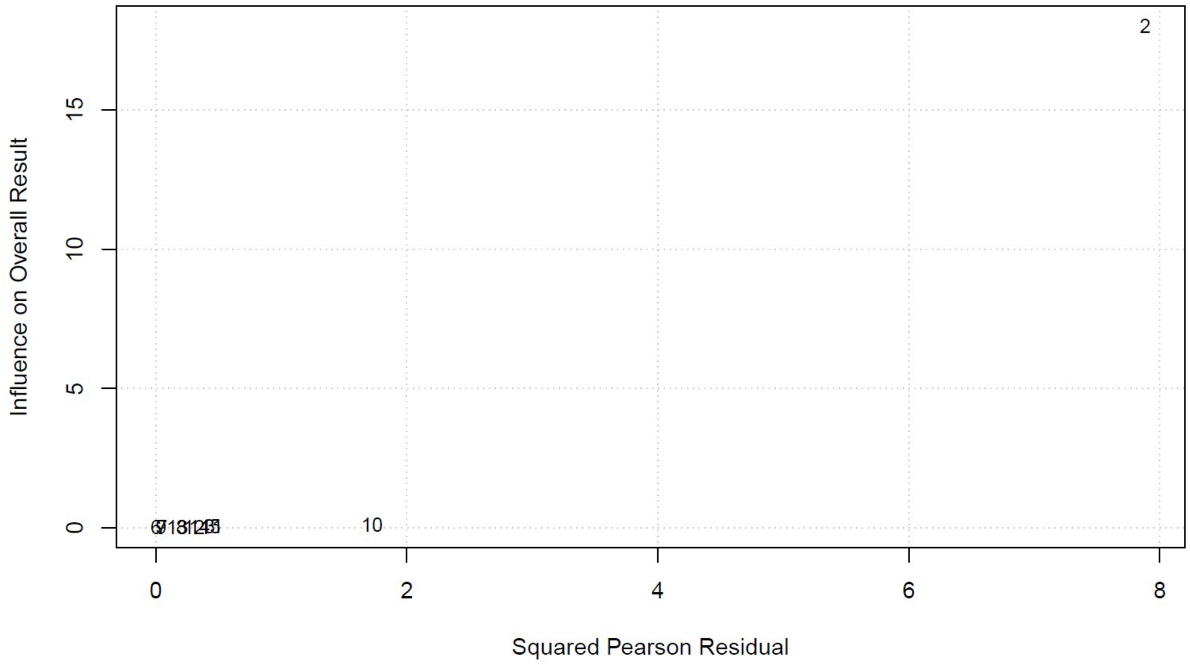

Figure S7; Baujat plot depicting outlier studies for associated environment for *Salmonella* spp.

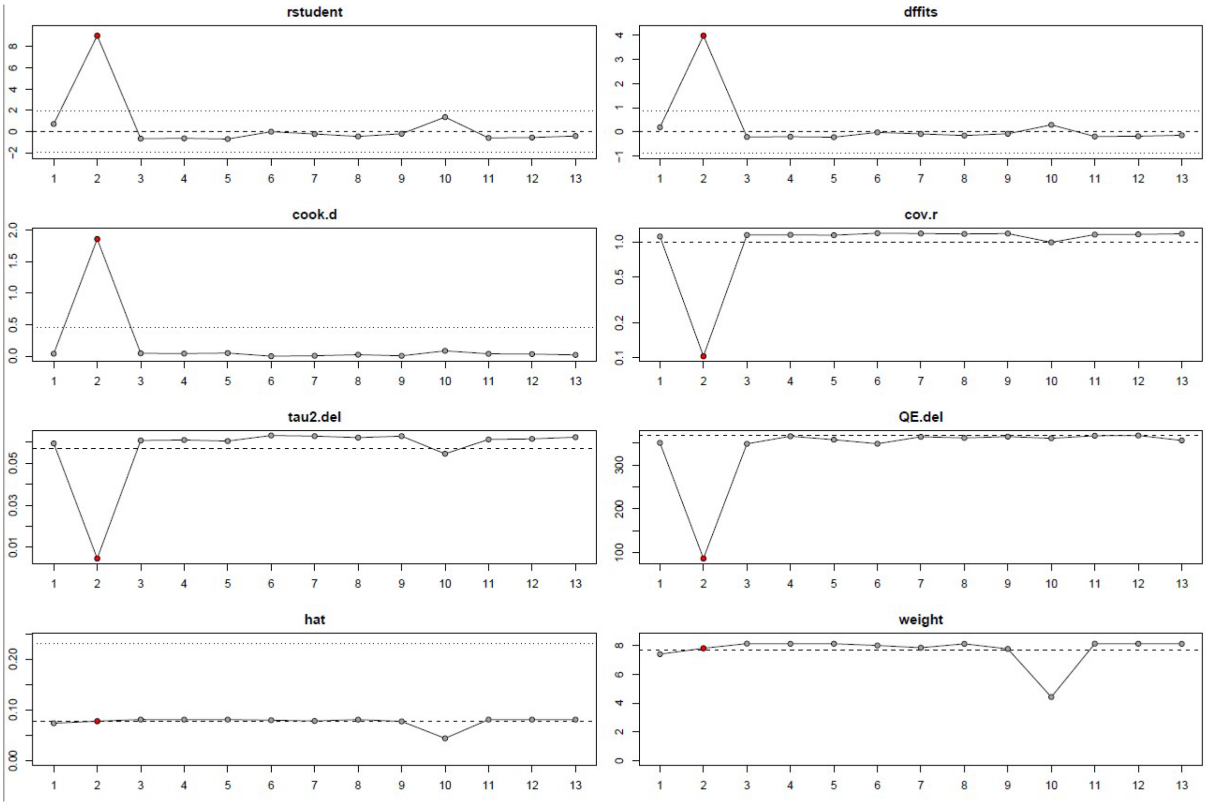

Figure S8; Plot depicting influential studies for associated environment for *Salmonella* spp.;

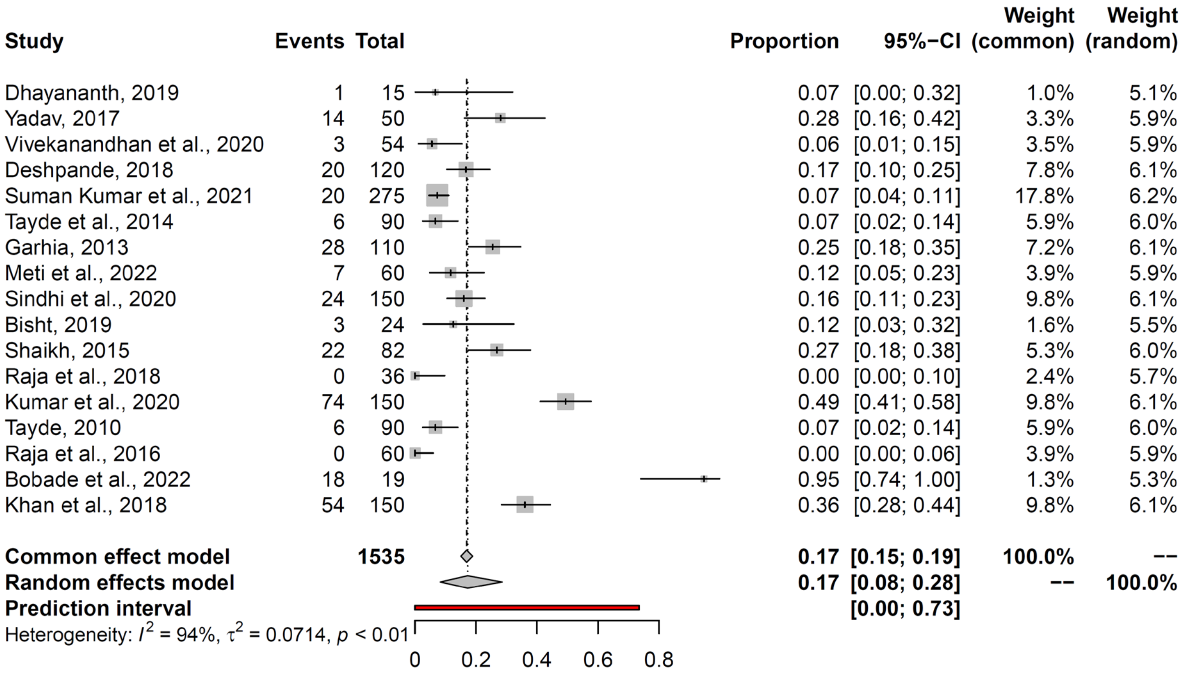

Figure S9; Forest plot depicting the pooled prevalence of *Campylobacter* spp. in retail chicken meat

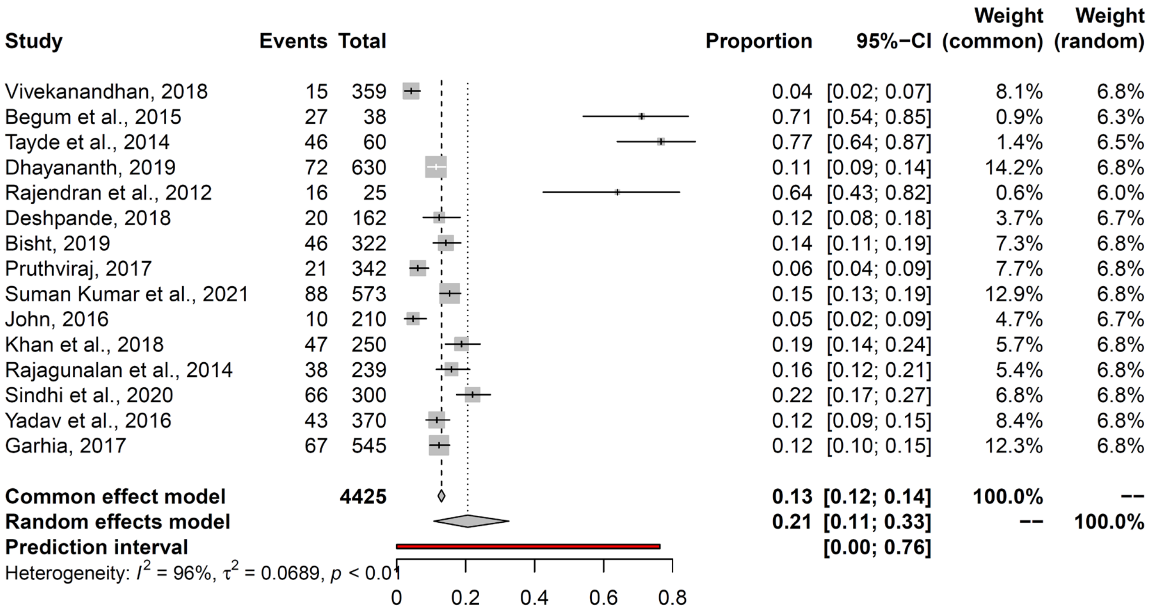

Figure S10; Forest plot depicting the pooled prevalence of *Campylobacter* spp. in the chicken-associated environment

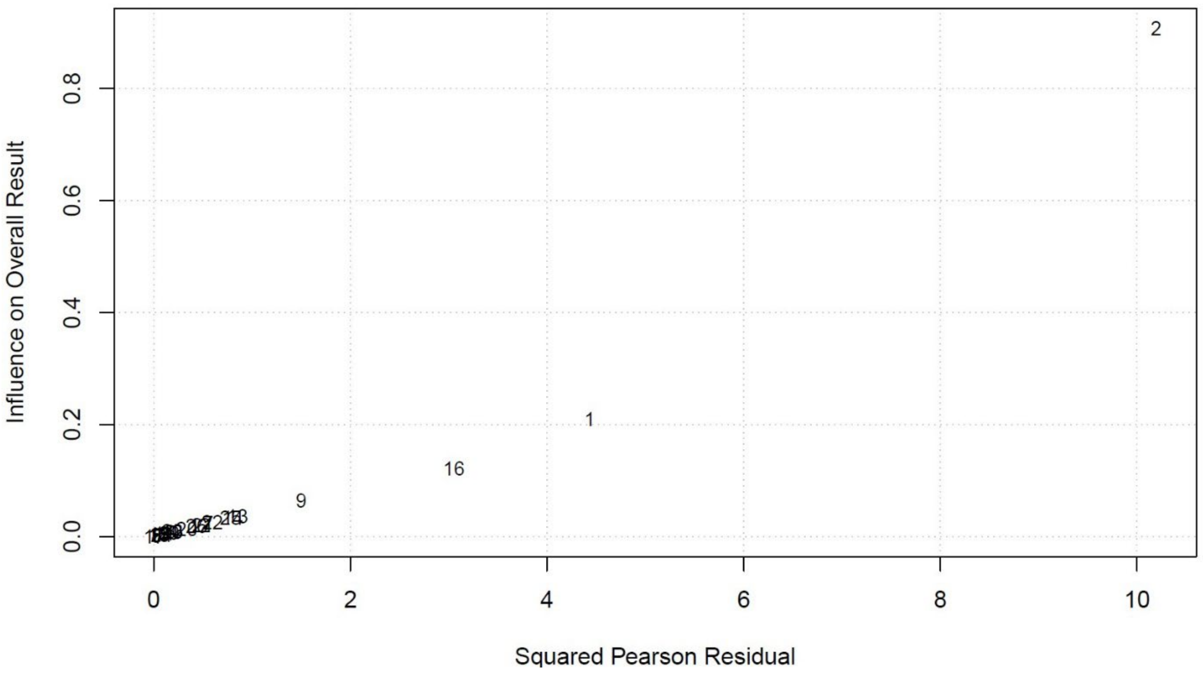

Figure S11; Baujat plot depicting outlier studies for retail chicken meat and associated environment for *Campylobacter* spp.

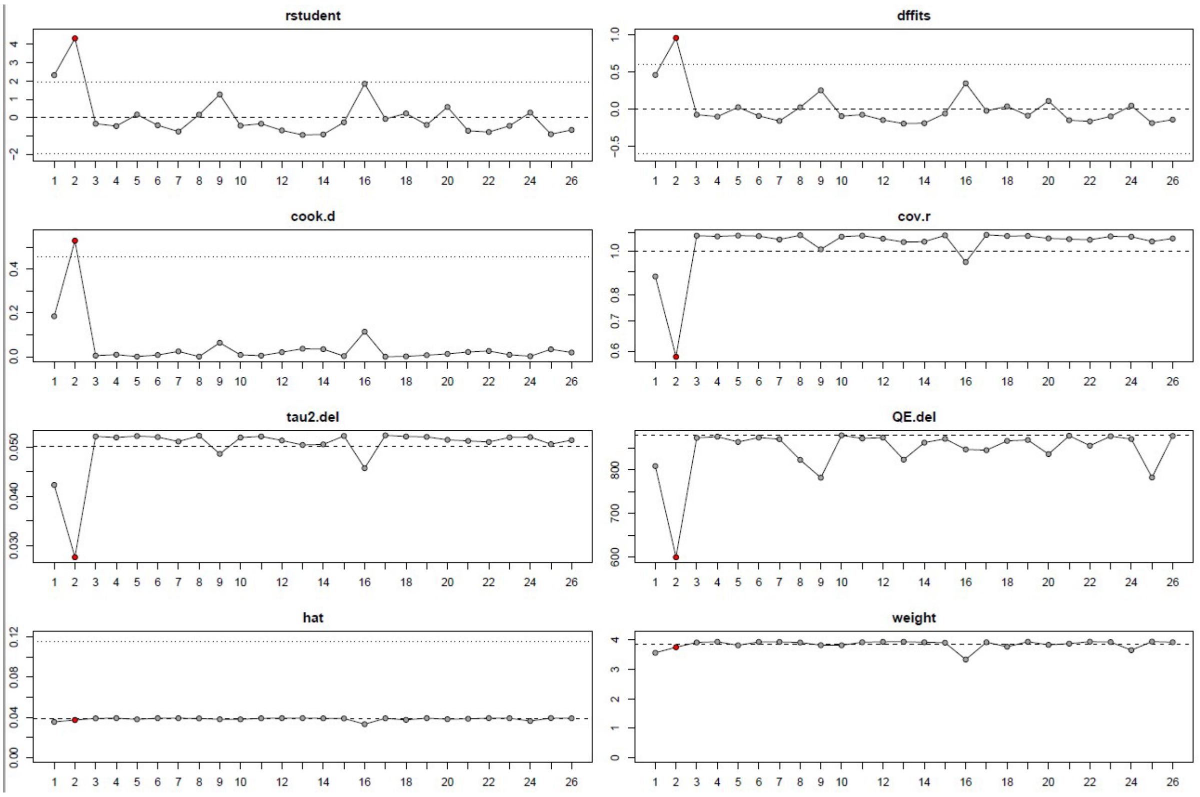

Figure S12; Plot depicting influential studies for retail chicken meat and associated environment for *Campylobacter* spp.

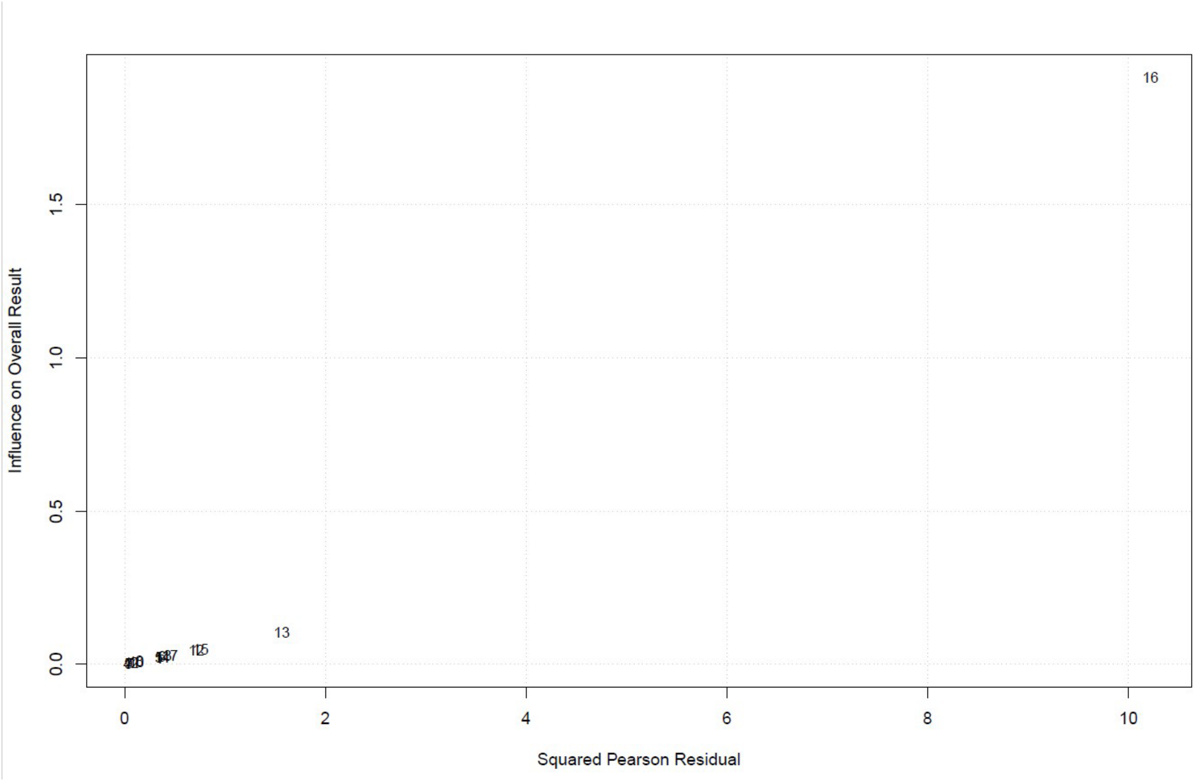

Figure S13; Baujat plot depicting outlier studies for retail chicken meat for *Campylobacter* spp.

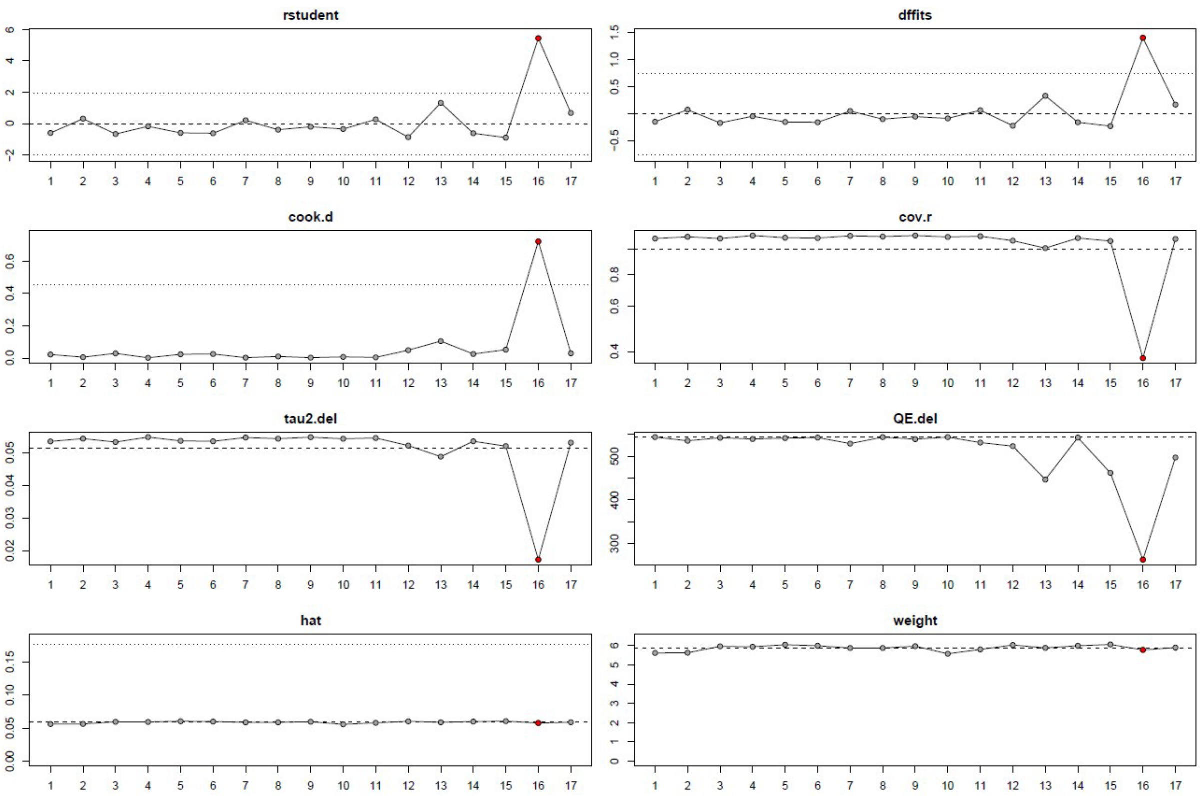

Figure S14; Plot depicting influential studies for retail chicken meat for *Campylobacter* spp.

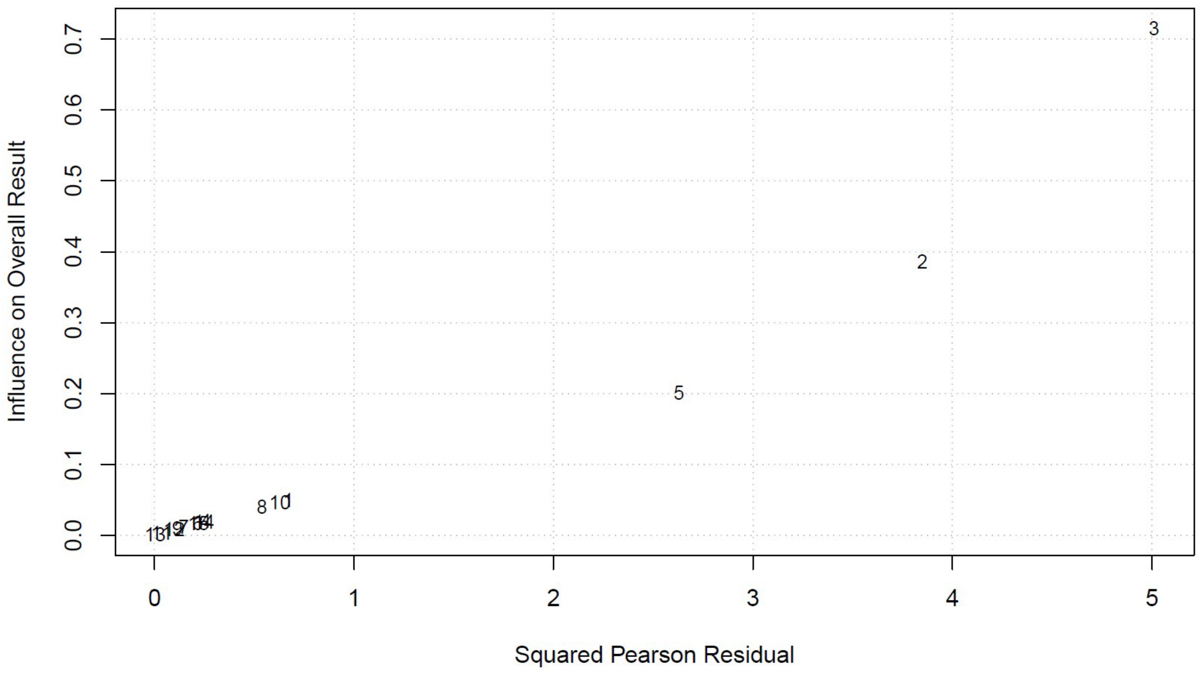

Figure S15; Baujat plot depicting outlier studies for associated environment for *Campylobacter* spp.

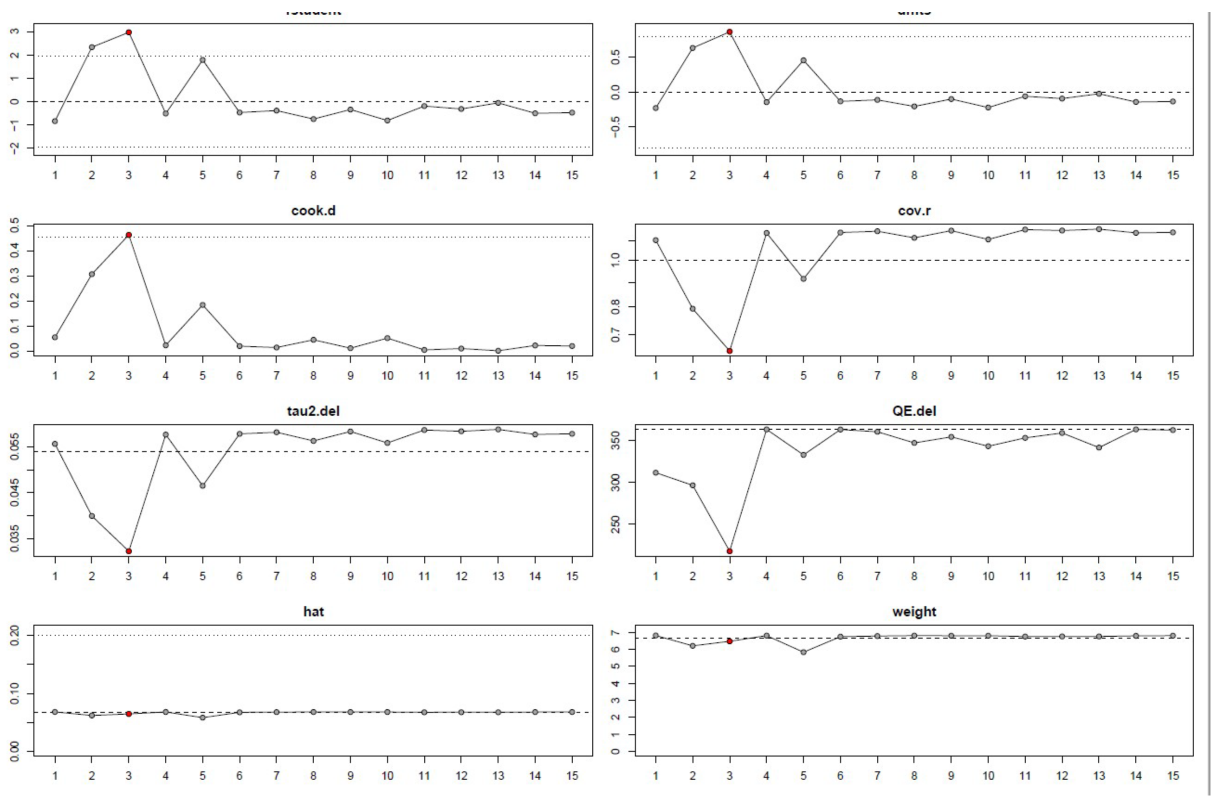

Figure S16; Plot depicting influential studies for associated environment for *Campylobacter* spp.

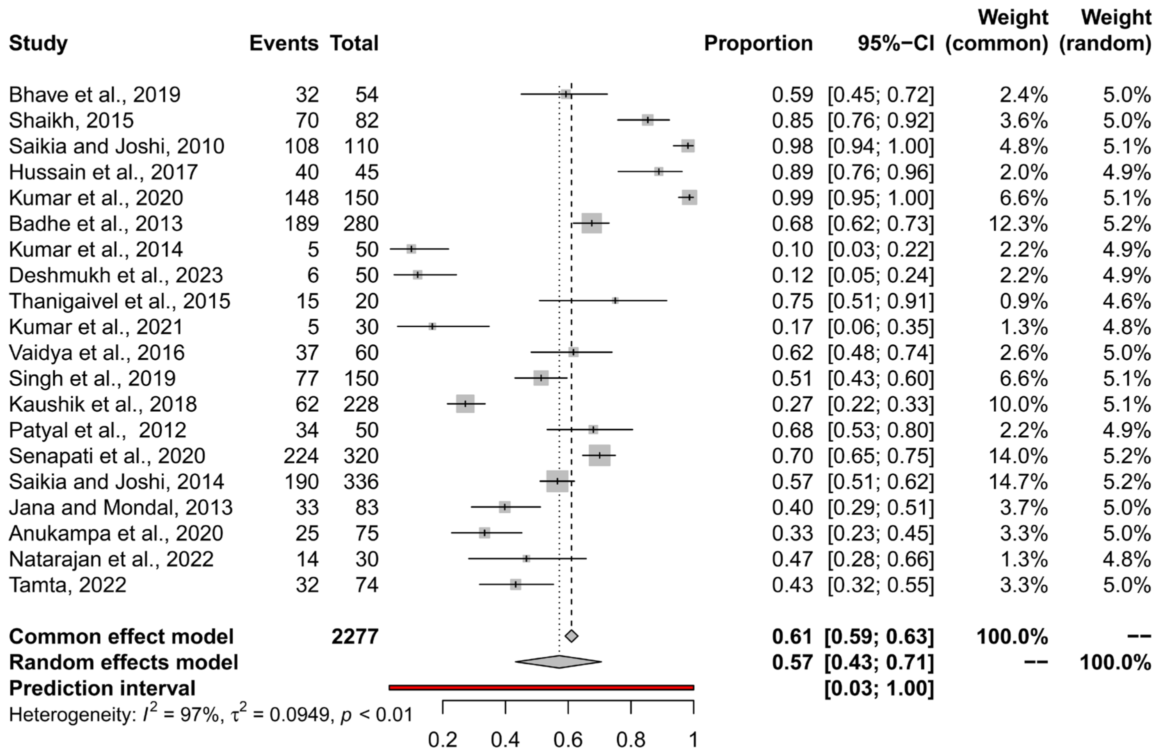

Figure S17; Forest plot depicting the pooled prevalence of *E. coli* in retail chicken meat

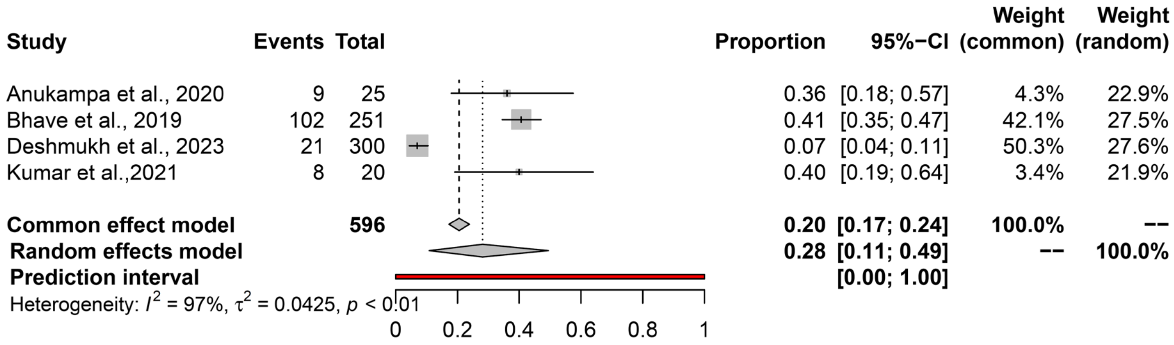

Figure S18; Forest plot depicting the pooled prevalence of *E. coli* in the chicken-associated environment

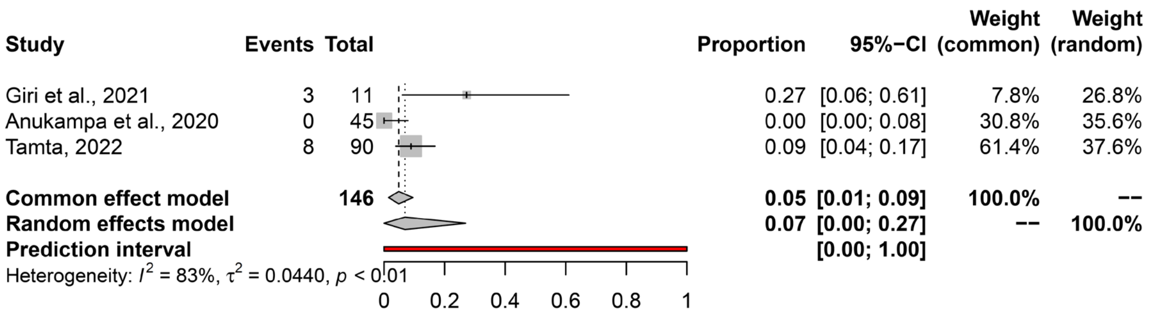

Figure S19; Forest plot depicting the pooled prevalence of *E. coli* in chicken meat products

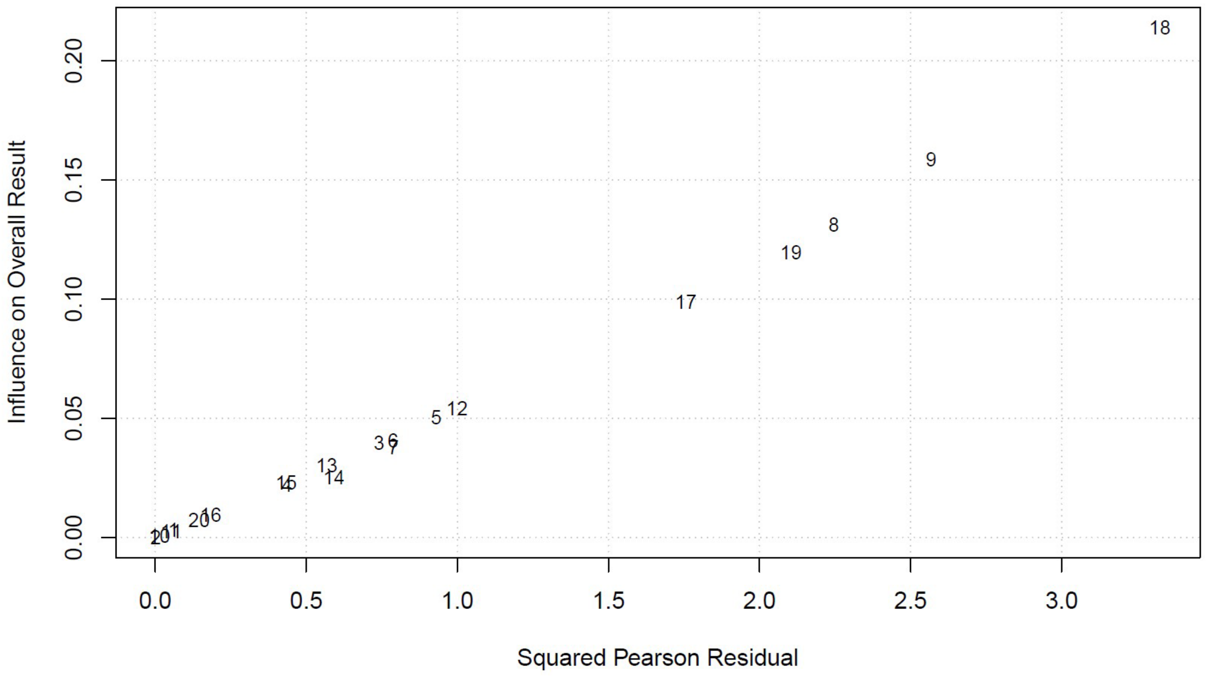

Figure S20; Baujat plot depicting outlier studies for retail chicken meat and associated environment for *E. coli*

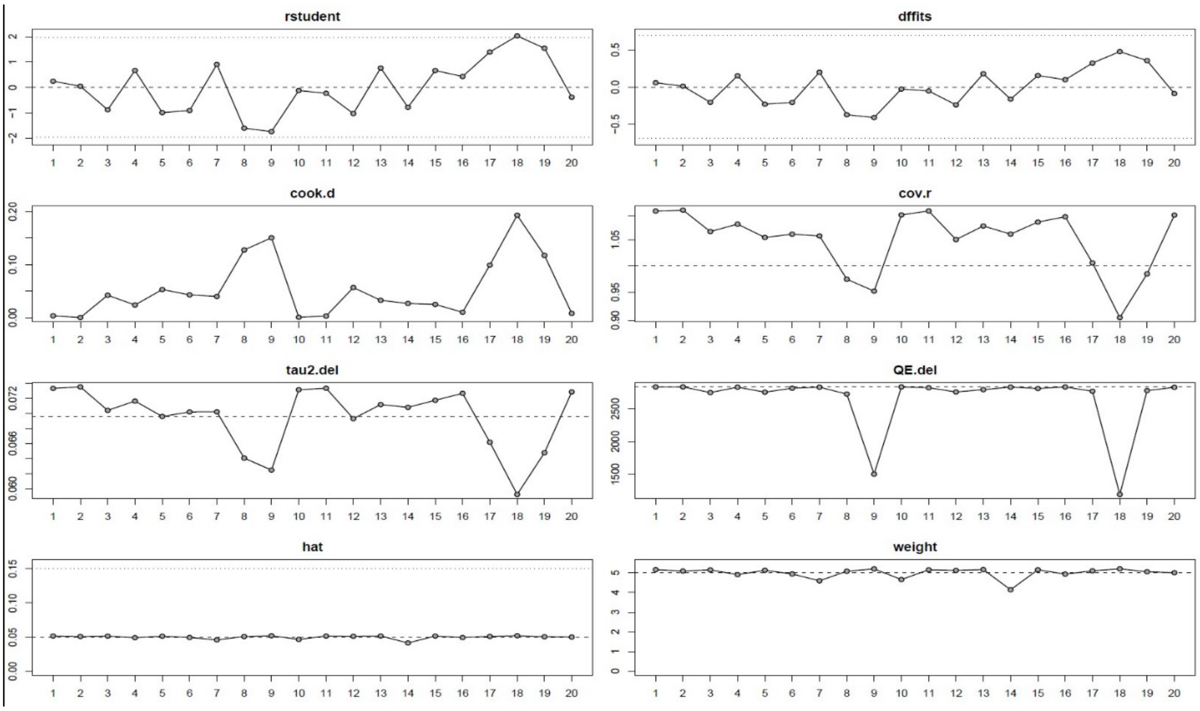

Figure S21; Plot depicting influential studies for retail chicken meat and associated environment for *E. coli*

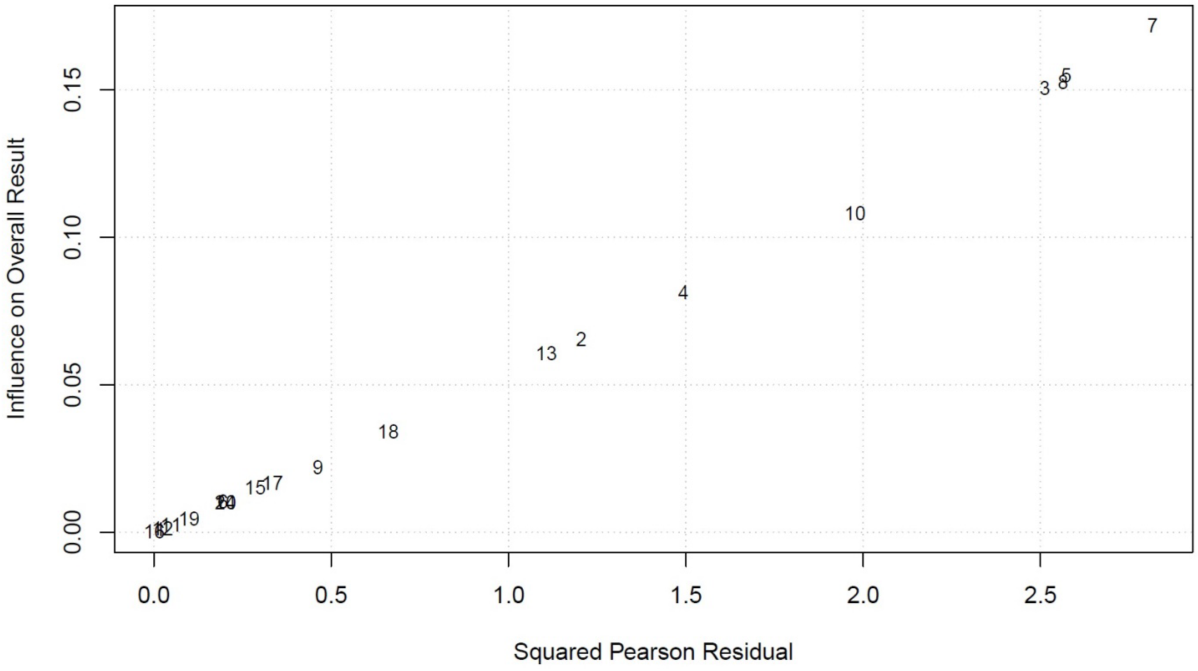

Figure S22; Baujat plot depicting outlier studies for retail chicken meat for *E. coli*

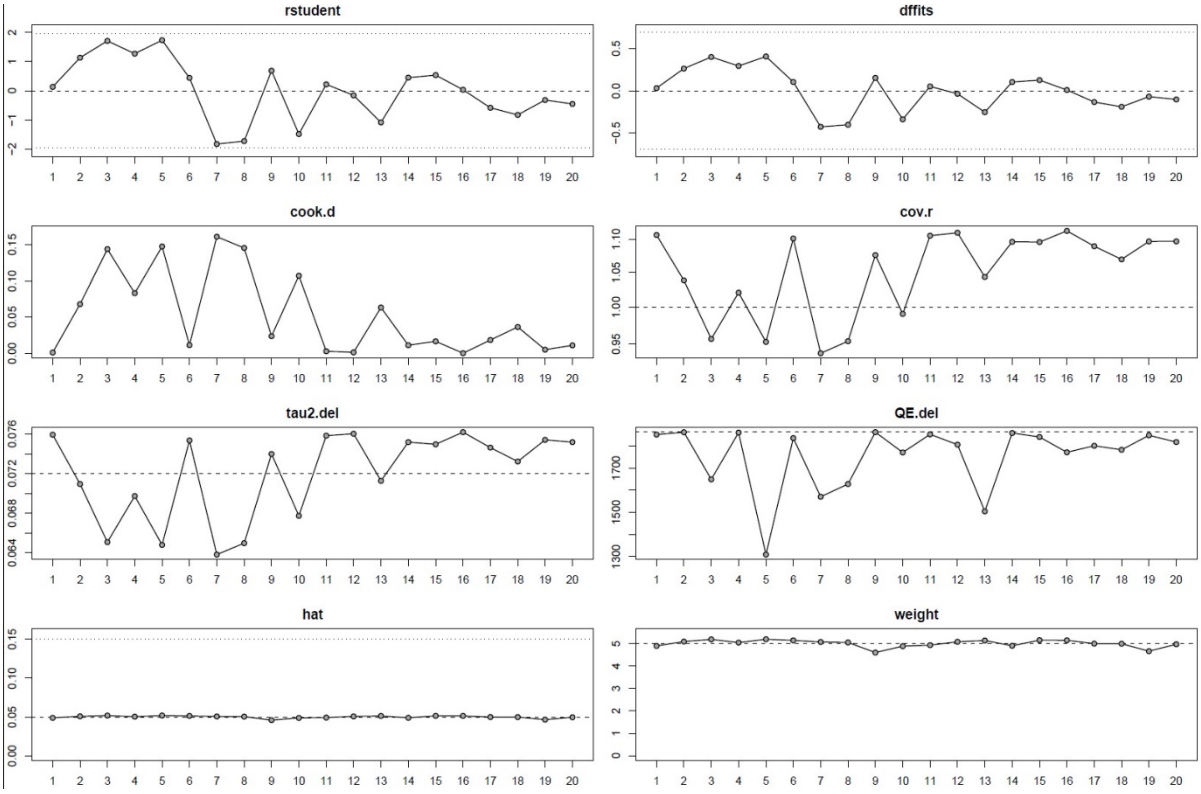

Figure S23; Plot depicting influential studies for retail chicken meat for *E. coli*

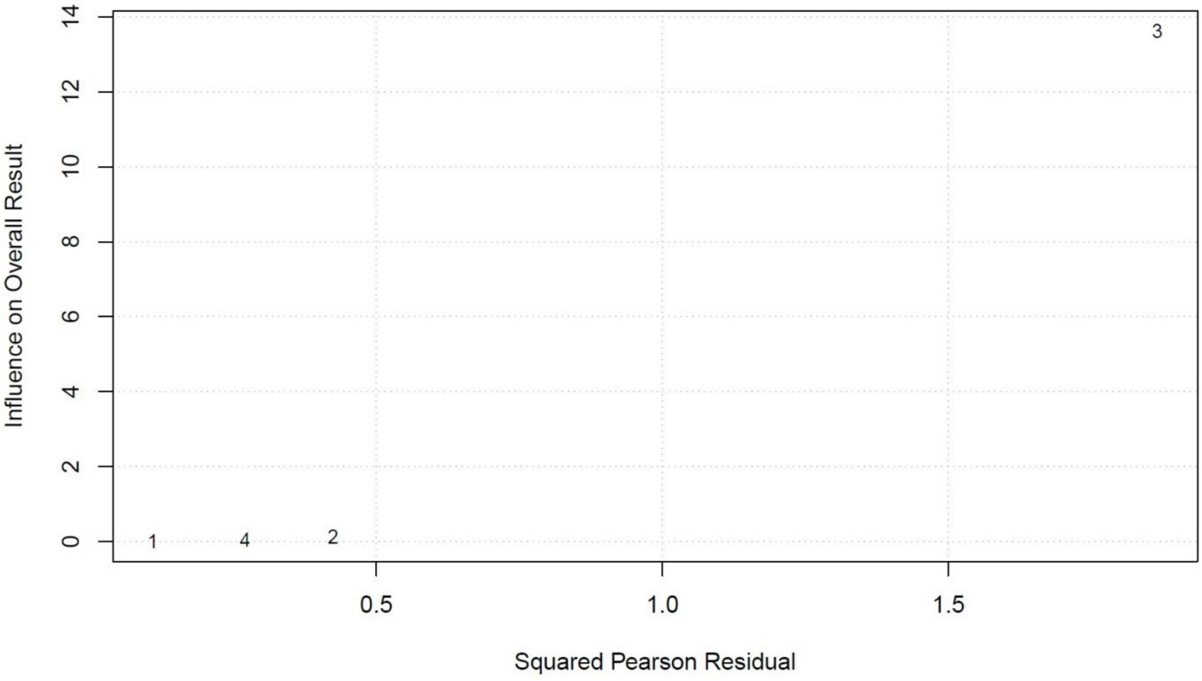

Figure S24; Baujat plot depicting outlier studies for associated environment for *E. coli*

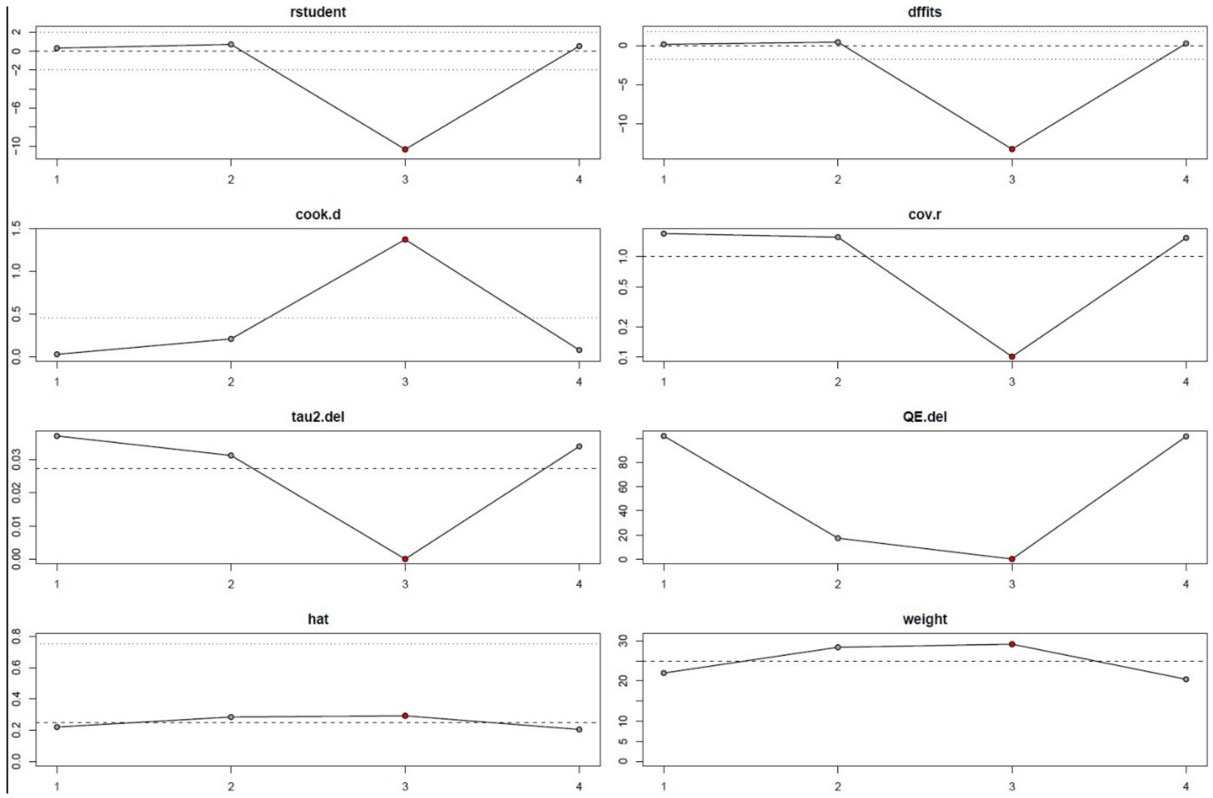

Figure S25; Plot depicting influential studies for associated environment for *E. coli*

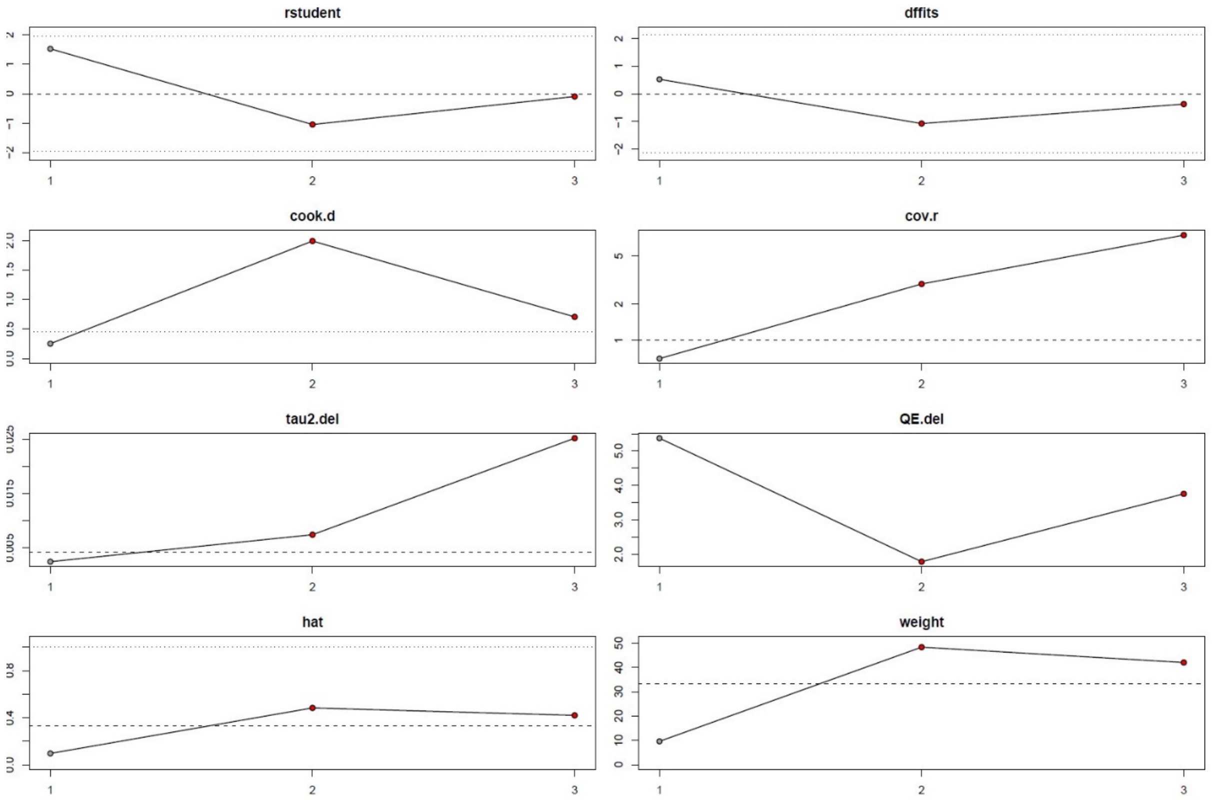

Figure S26; Plot depicting influential studies for chicken meat products for *E. coli*

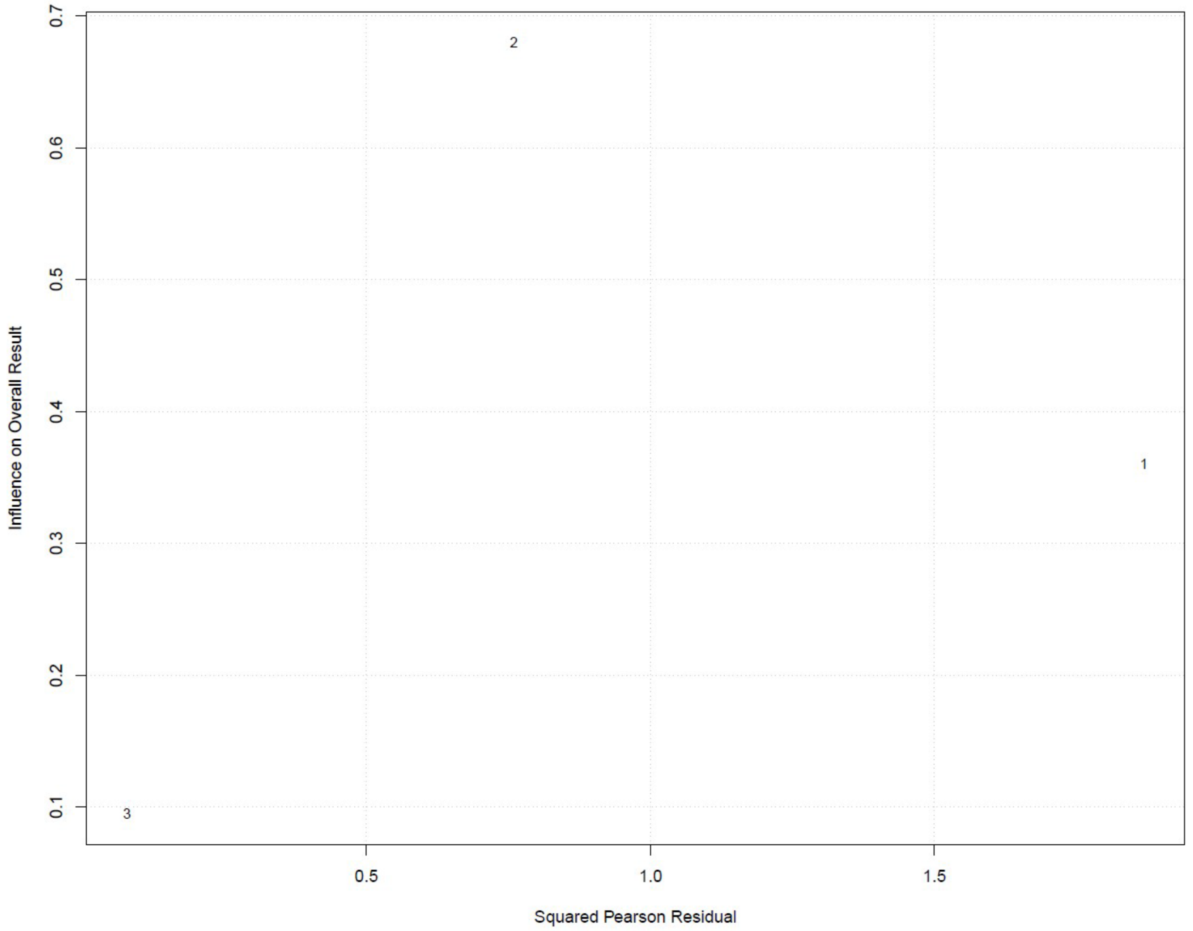

Figure S27; Baujat plot depicting outlier studies for chicken meat products for *E. coli*

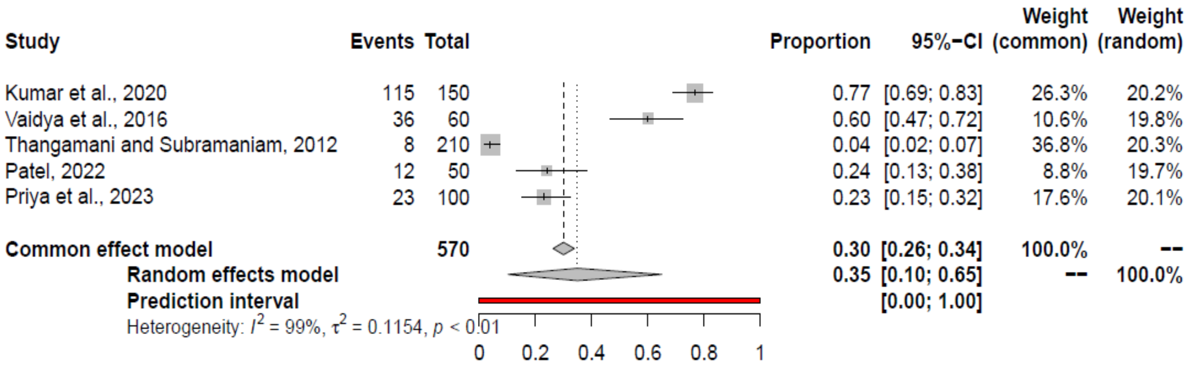

Figure S28; Forest plot depicting the pooled prevalence of *C. perfringens* in retail chicken meat

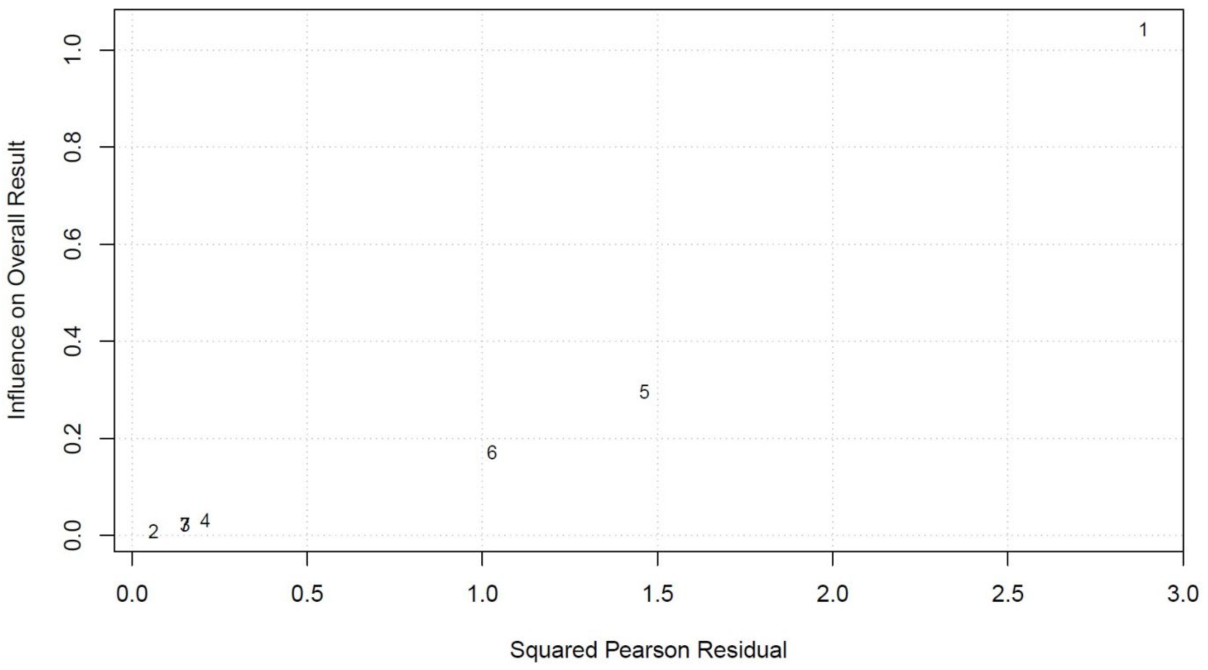

Figure S29; Baujat plot depicting outlier studies for retail chicken meat and associated environment for *C. perfringens*

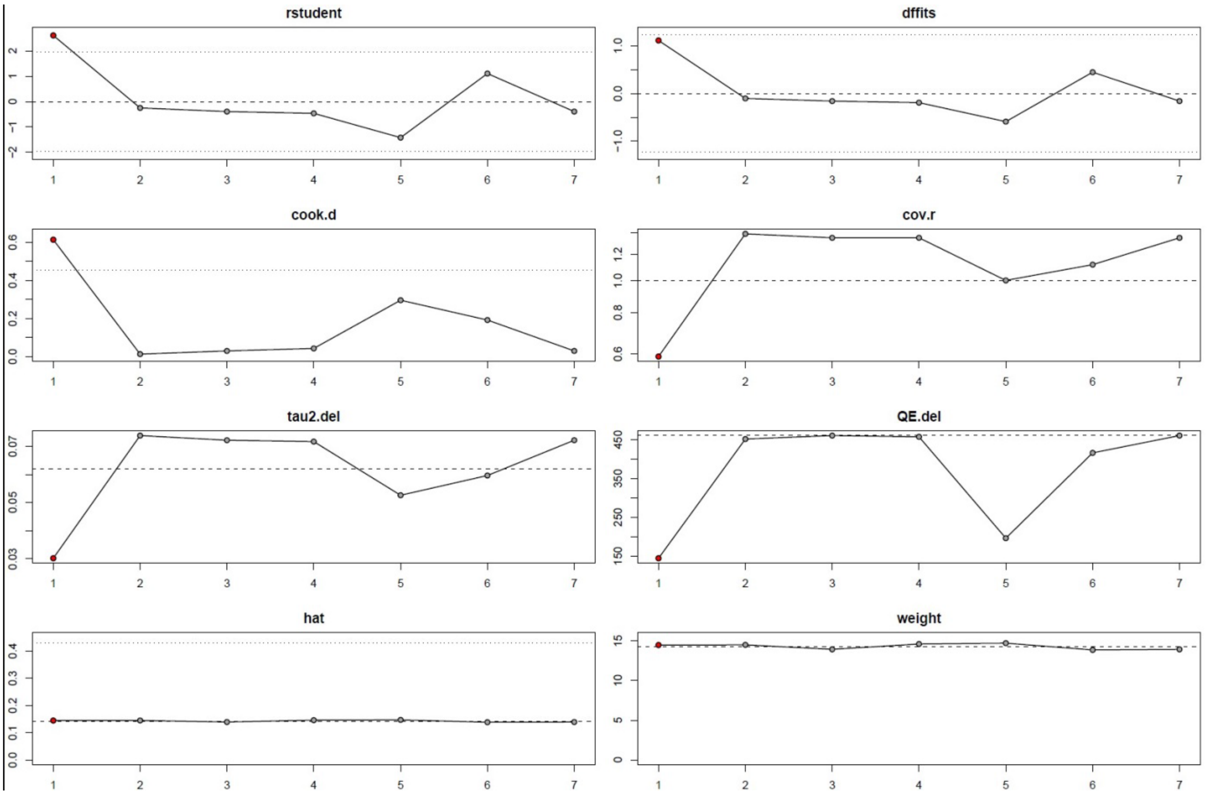

Figure S30; Plot depicting influential studies for retail chicken meat and associated environment for *C. perfringens*

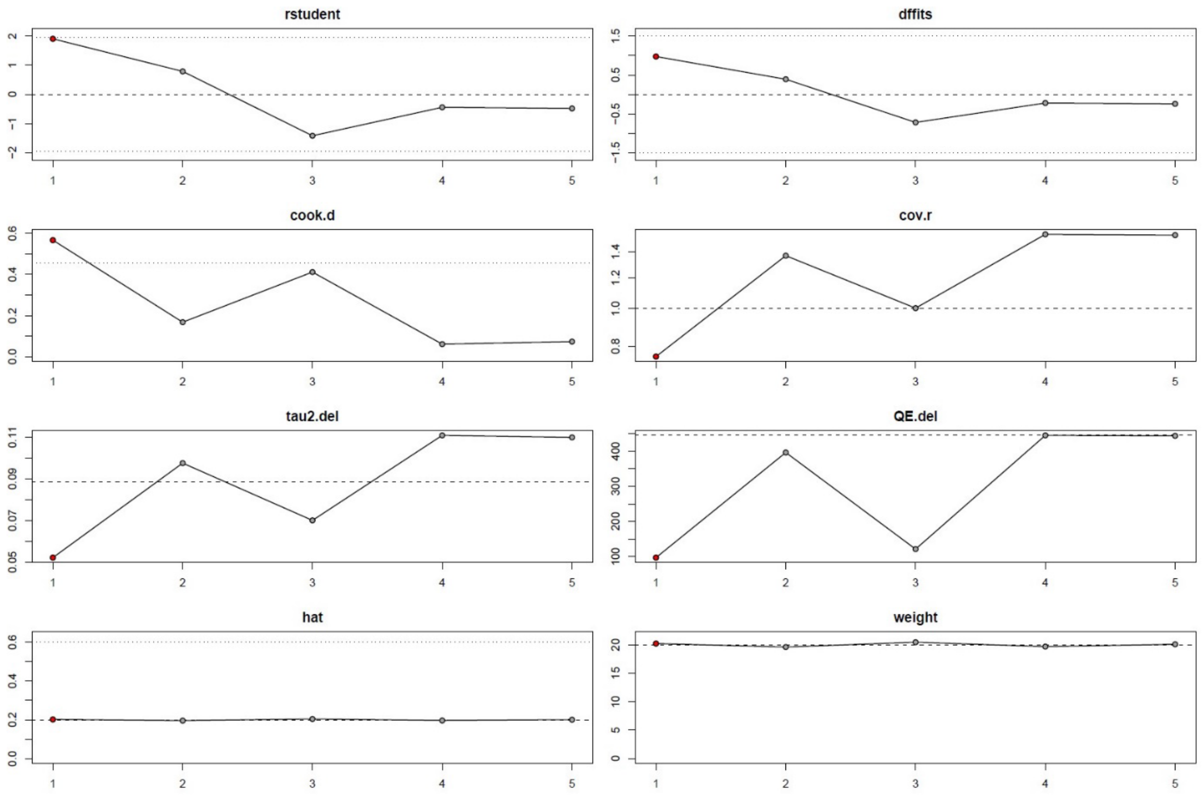

Figure S31; Plot depicting influential studies for retail chicken meat for *C. perfringens*

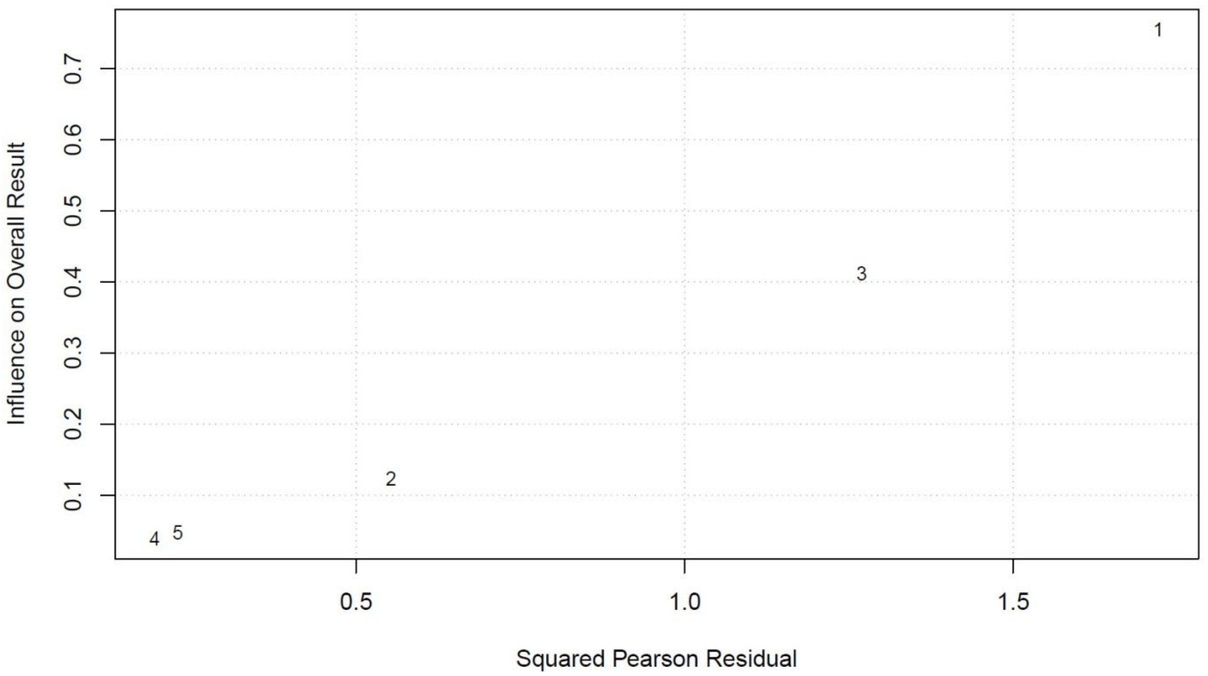

Figure S32; Baujat plot depicting outlier studies for retail chicken meat for *C. perfringens*

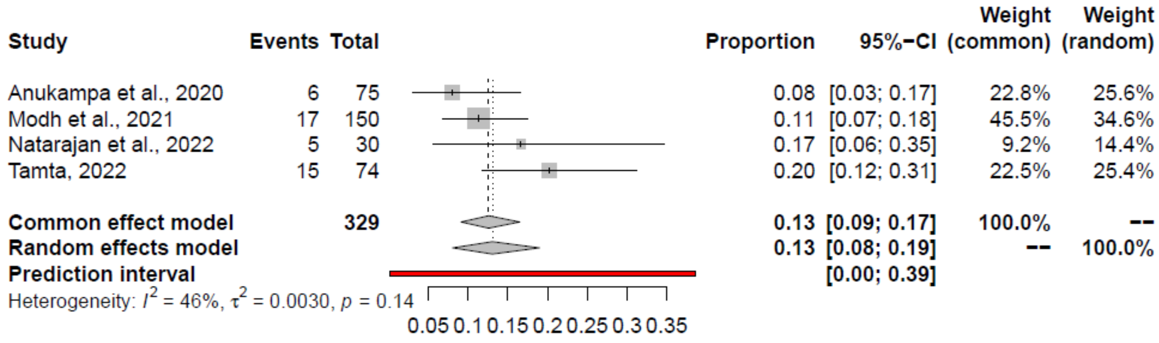

Figure S33; Forest plot depicting the pooled prevalence of *K. pneumoniae* in retail chicken meat

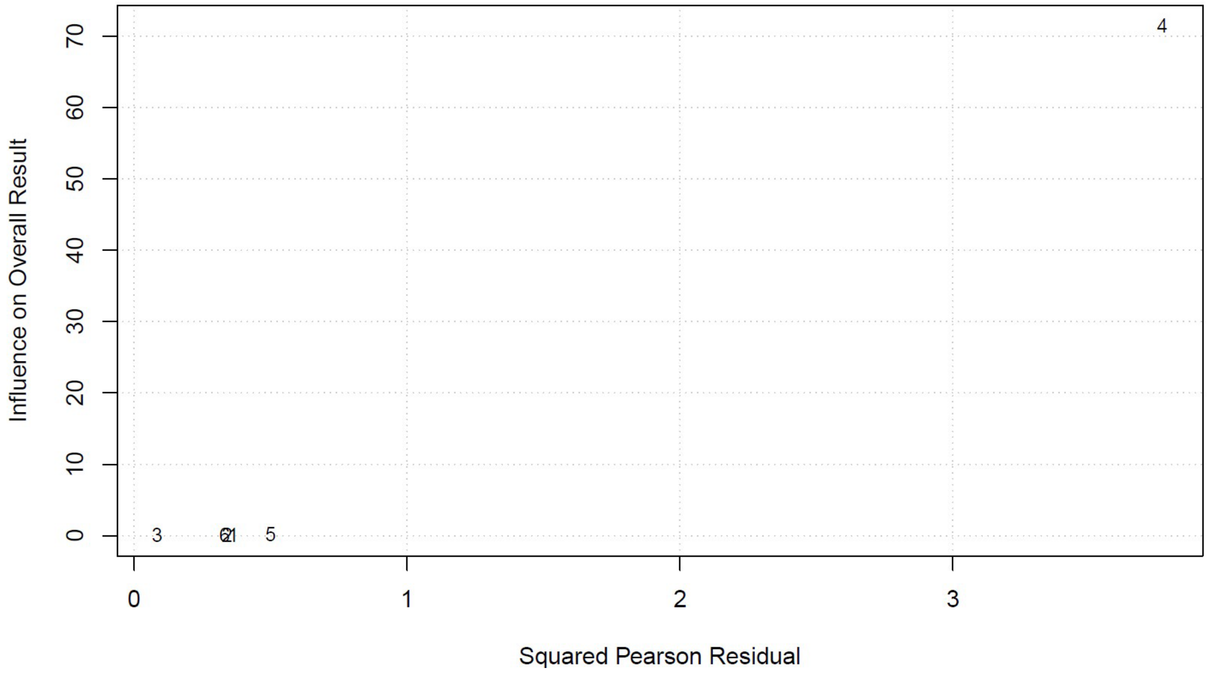

Figure S34; Baujat plot depicting outlier studies for retail chicken meat and associated environment for *K. pneumoniae*

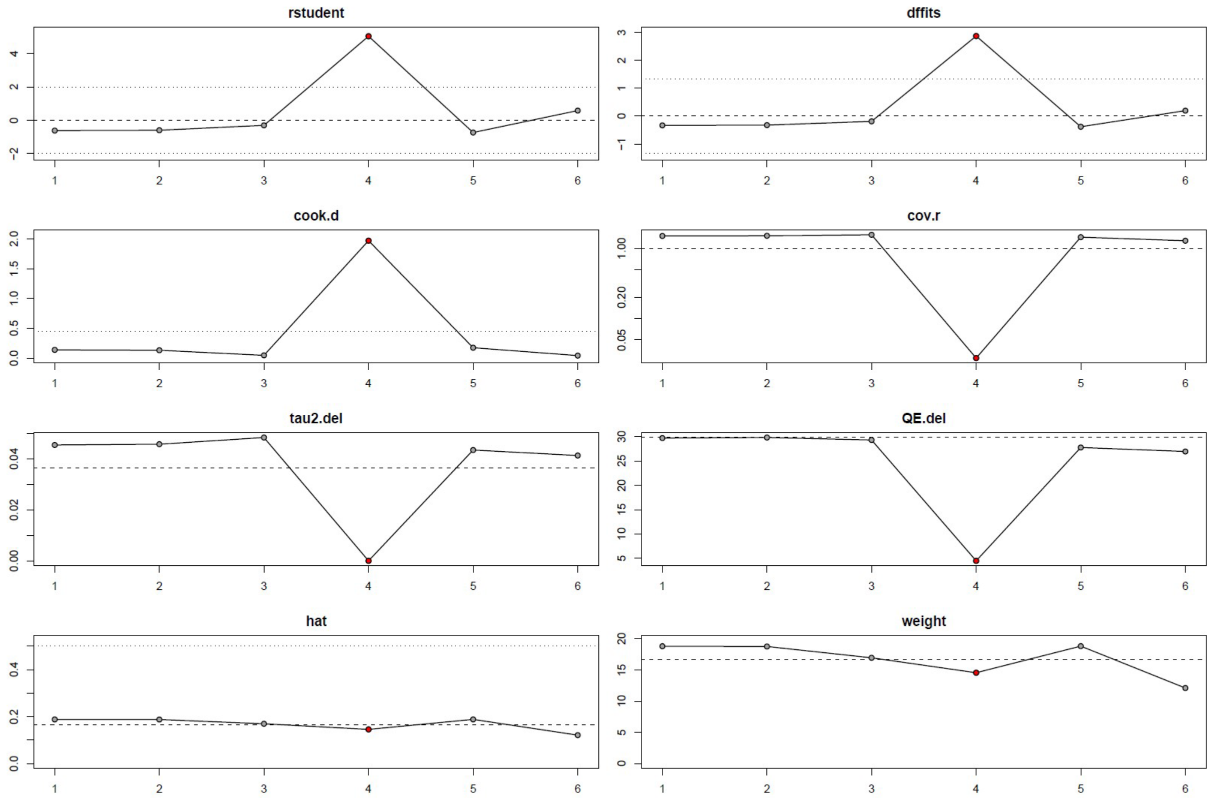

Figure S35; Plot depicting influential studies for retail chicken meat and associated environment for *K. pneumoniae*

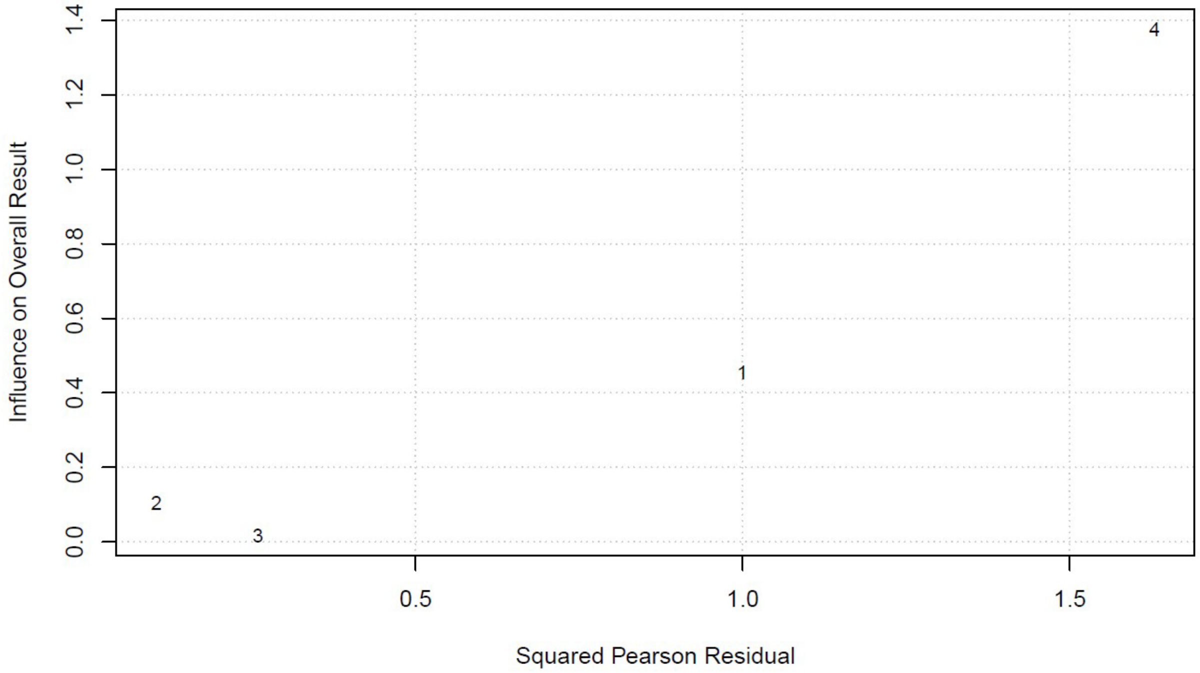

Figure S36; Baujat plot depicting outlier studies for retail chicken meat for *K. pneumoniae*

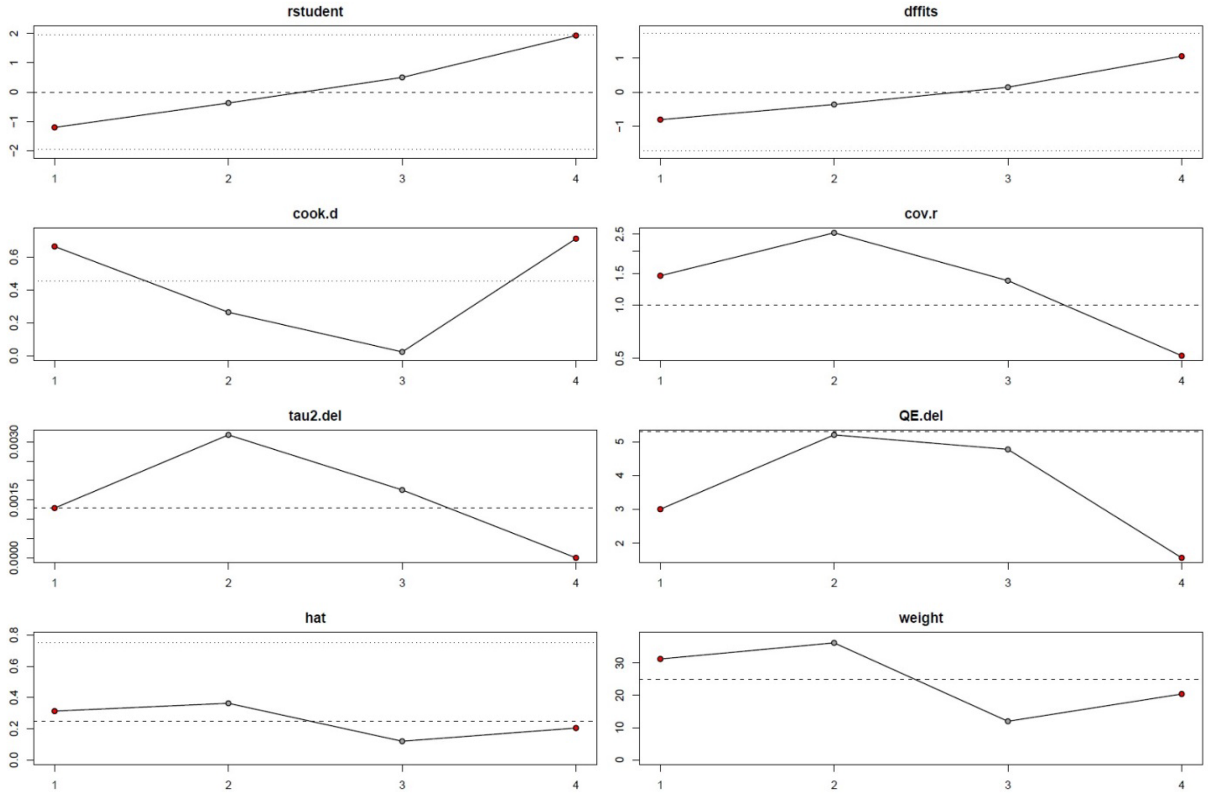

Figure S37; Plot depicting influential studies for retail chicken meat for *K. pneumoniae*

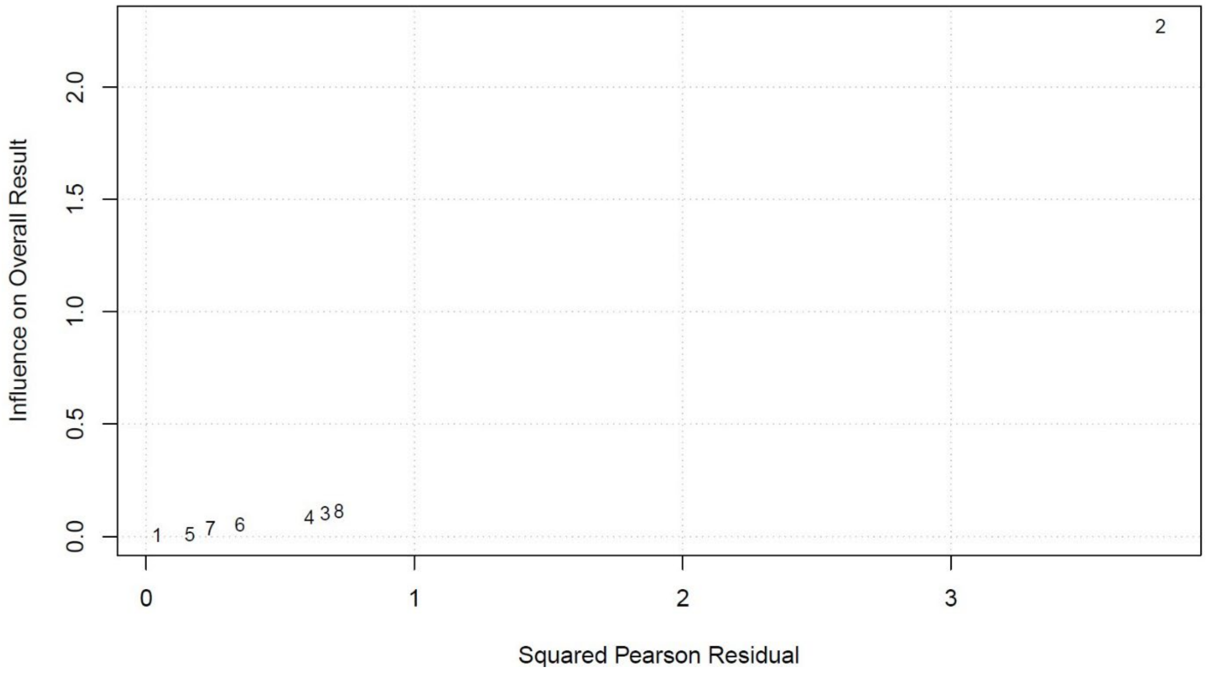

Figure S38; Baujat plot depicting outlier studies for retail chicken meat and associated environment for *Listeria* spp.

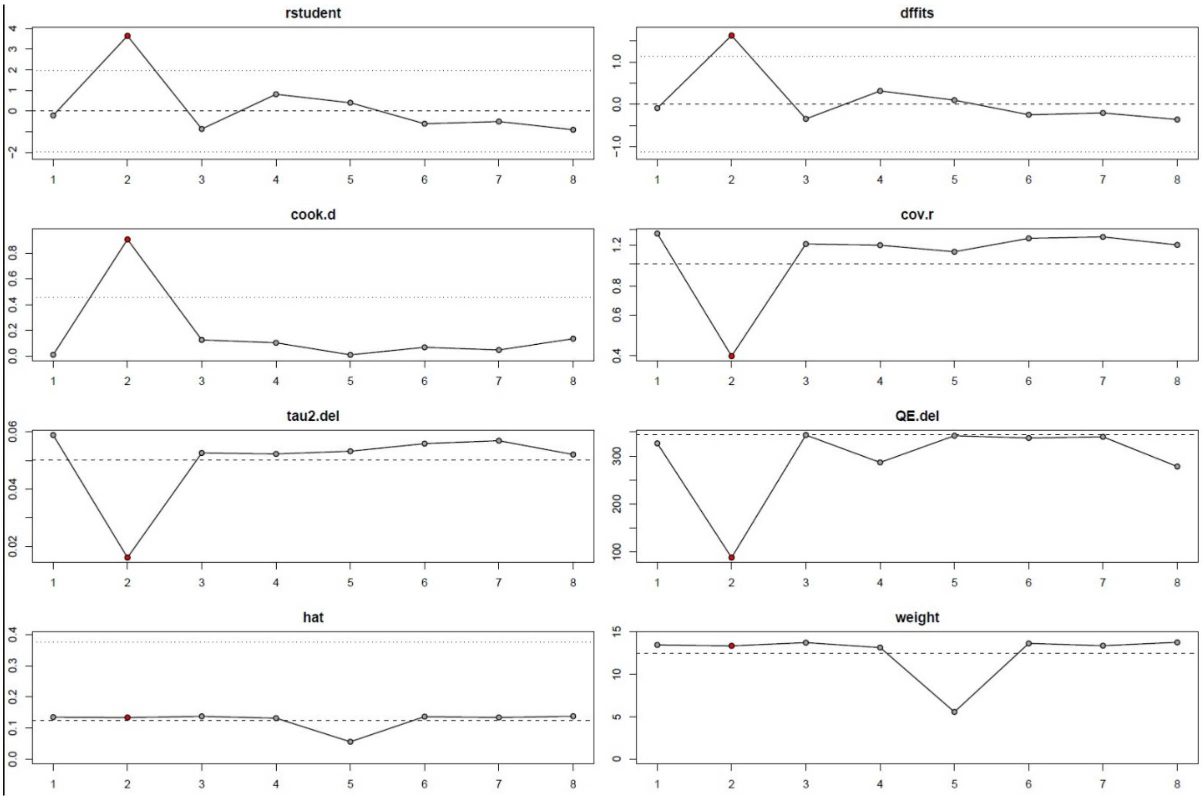

Figure S39; Plot depicting influential studies for retail chicken meat and associated environment for *Listeria* spp.

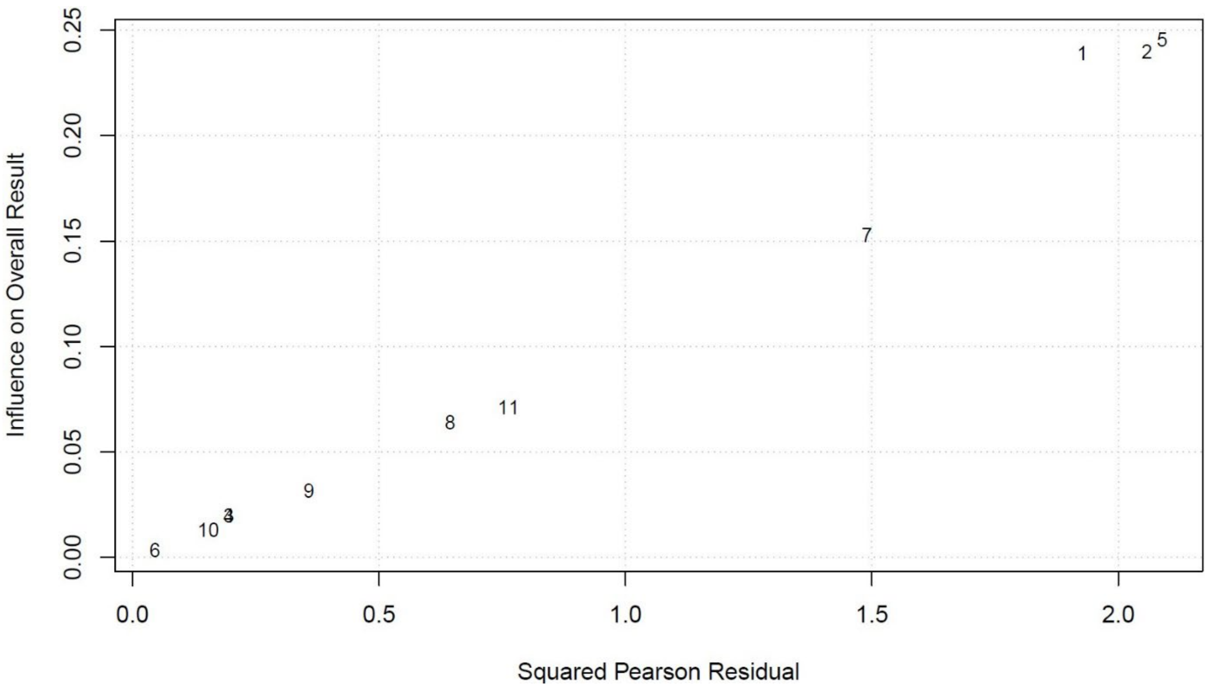

Figure S40; Baujat plot depicting outlier studies for retail chicken meat and associated environment for *S. aureus*

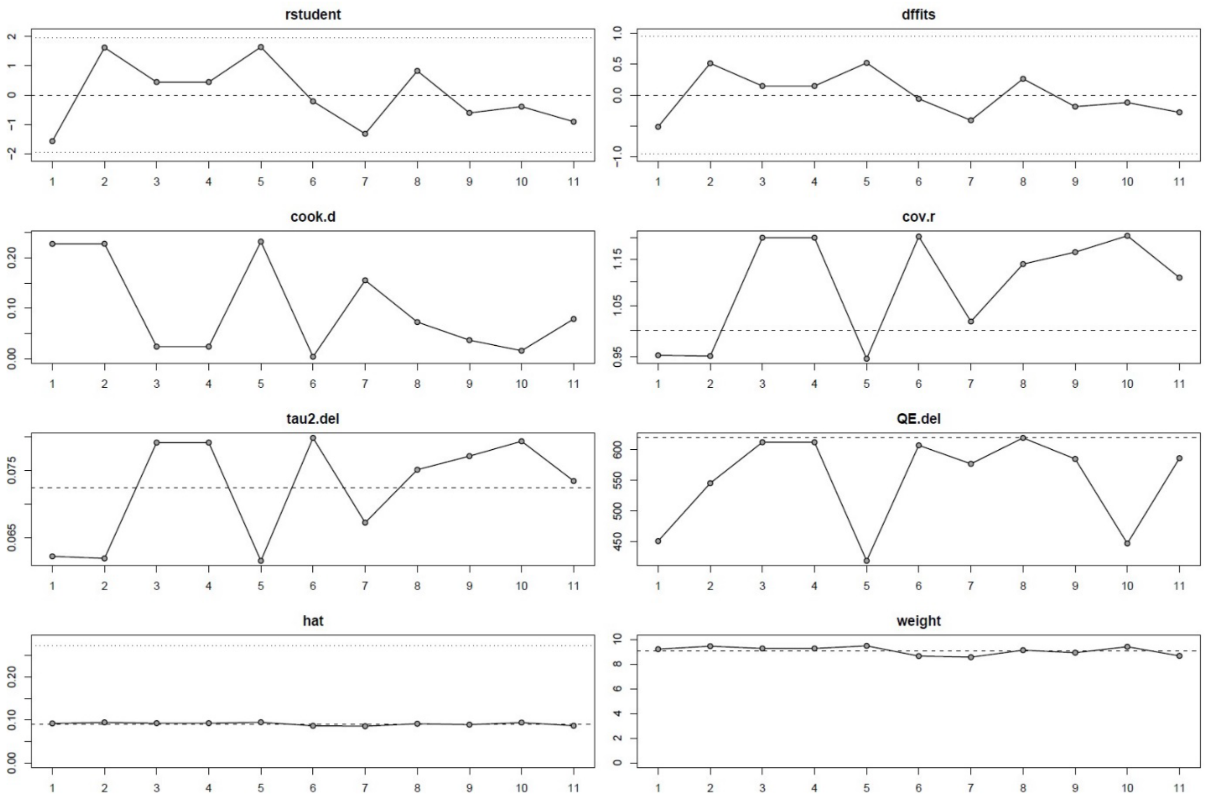

Figure S41; Plot depicting influential studies for retail chicken meat and associated environment for *S. aureus*.
